# Hypoxia‐Induced PRMT1 Lactylation Drives Vimentin Arginine Asymmetric Dimethylation in Tumor Metastasis

**DOI:** 10.1002/advs.202509861

**Published:** 2025-08-30

**Authors:** Jia Zhou, Shuying Qiu, Xia Yang, Yan Wu, Xinxia Yao, Hangqi Hu, Jingfeng Luo, Chandra Sugiarto Wijaya, Lingfeng Ma, Xiaojun Long, Lingna Xu, Jinquan Liu, Chaoqun Wang, Yibin Pan, Xiaona Chen, Hongchuan Jin, Xian Wang

**Affiliations:** ^1^ Department of Medical Oncology, Cancer Center of Zhejiang University, Sir Run Run Shaw Hospital, School of Medicine Zhejiang University Hangzhou Zhejiang 310016 China; ^2^ Department of pathology Sir Run Run Shaw Hospital, School of Medicine Zhejiang University Hangzhou Zhejiang 310016 China; ^3^ Department of Colorectal Surgery, Key Laboratory of Biological Treatment of Zhejiang Province, Sir Run Run Shaw Hospital, School of Medicine Zhejiang University Hangzhou Zhejiang 310016 China; ^4^ Department of Hepatobiliary and Pancreatic Surgery and Zhejiang Provincial Key Laboratory of Pancreatic Disease, The First Affiliated Hospital Zhejiang University School of Medicine Hangzhou Zhejiang 310029 China; ^5^ Department of Pathology Affiliated Dongyang Hospital of Wenzhou Medical University Dongyang Zhejiang 322100 China; ^6^ Department of Obstetrics and Gynecology, Sir Run Run Shaw Hospital, School of Medicine Zhejiang University Hangzhou Zhejiang 310016 China

**Keywords:** arginine methylation, cancer metastasis, hypoxia, lactylation, PRMT1, vimentin

## Abstract

Metastasis contributes to around 90% of cancer mortality, but effective strategies to disrupt metastatic cascades remain elusive. Hypoxia‐driven epithelial‐mesenchymal transition (EMT) promotes cancer cell spread, yet the post‐translational mechanisms governing cytoskeletal reprogramming here remain incompletely defined. This study reports a hypoxia‐inducible post‐translational modification cascade: under hypoxia, protein arginine methyltransferase 1 (PRMT1) is lactylated at evolutionarily conserved residues K134/K145, enhancing its methyltransferase activity to catalyze the asymmetric dimethylation (aDMA) of vimentin at R64. This modification drives vimentin filament assembly, cytoskeletal remodeling, and metastasis in preclinical models. shPRMT1 or vimentin R64K mutation (methylation‐deficient) abrogates hypoxia‐enhanced migration in vitro and metastasis in vivo. Hypoxia reduces the protein levels of HDAC8 (PRMT1’s delactylase), boosting PRMT1 lactylation. PRMT1 K134R/K145R mutants (lactylation ‐ deficient) lose the ability to bind vimentin and fail to rescue filament formation. In triple‐negative breast cancer (TNBC), vimentin R64 aDMA levels correlate with advanced tumor stage and poor patient survival. PRMT1 inhibitor MS023 reduces xenograft metastasis with low toxicity. These findings establish a hypoxia‐PRMT1‐vimentin axis, identifying vimentin R64 aDMA as a metastatic regulator. Inhibiting PRMT1 represents a promising anti‐metastasis strategy.

## Graphical abstract

Hypoxia triggers lactylation of PRMT1 at K134/K145, enhancing its methyltransferase activity to asymmetrically dimethylate vimentin R64. This post‐translational cascade promotes vimentin filament assembly, cytoskeletal remodeling, and tumor metastasis, with PRMT1 inhibition (MS023) reducing metastatic colonization. Clinically, vimentin R64 asymmetric dimethylation correlates with advanced TNBC stage and poor survival, highlighting a targetable metastatic axis.

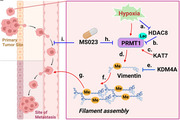

## 1 Introduction

Metastasis accounts for nearly 90% of cancer‐related deaths^[^ ^1^ ^]^; however, effective therapeutic strategies for the prevention of metastasis remain elusive. Clinically detectable metastatic lesions are typically associated with poor prognosis and are largely refractory to current treatments. Consequently, considerable research efforts have been devoted to uncovering the biological mechanisms underlying tumor dissemination and to improving therapeutic outcomes in patients with advanced‐stage malignancies.^[^ ^2^ ^]^ Hypoxia, a pathophysiological hallmark of solid tumors, is observed in both subcutaneous xenograft cores and heterogeneous regions of autochthonous tumors,^[^ ^3^ ^]^ and drives cancer progression through multiple pathways, including angiogenesis, metabolic reprogramming, and metastatic dissemination.^[^ ^4^ ^]^ This oxygen‐deprived microenvironment stabilizes hypoxia‐inducible factors (HIFs), with clinical studies demonstrating that HIF‐1α overexpression in primary tumors correlates with increased metastatic risk, disease recurrence, and mortality.^[^ ^3^ ^]^ Nevertheless, the precise molecular mechanisms connecting hypoxic stress to metastatic initiation require further investigation.

The epithelial‐mesenchymal transition (EMT) is a fundamental biological process in cancer metastasis. During EMT, polarized epithelial cells acquire mesenchymal characteristics through loss of cell‐cell adhesion and apical‐basal polarity.^[^ ^5^ ^]^ Vimentin, a type III intermediate filament protein, serves as a biomarker of aggressive phenotypes across multiple carcinomas.^[^ ^6^ ^]^ Clinical evidence from breast, lung, esophageal, gastric, and colorectal cancers consistently demonstrates positive correlations between vimentin expression and metastatic progression(6). Diverse post‐translational modifications (PTMs), including phosphorylation,^[^ ^7^ ^]^ S‐glutathionylation,^[^ ^8^ ^]^ acetylation,^[^ ^9^ ^]^ ubiquitination,^[^ ^10^ ^]^ SUMOylation,^[^ ^11^ ^]^ and ADP ribosylation,^[^ ^12^ ^]^ are involved in regulating vimentin function. For example, S‐glutathionylation impedes filament elongation,^[^ ^8^ ^]^ while PIAS1‐mediated SUMOylation enhances cell migration.^[^ ^11^ ^]^ Recent studies have revealed that PRMT5‐mediated symmetric dimethylarginine (sDMA) regulates vimentin stability in MTAP‐deficient lung cancer.^[^ ^13^ ^]^ In this study, we identified a novel regulatory mechanism involving PRMT1‐dependent asymmetric dimethylation (aDMA) at previously uncharacterized arginine residue within vimentin. This discovery raises the question of whether vimentin aDMA modulates EMT‐mediated metastatic progression.

Protein arginine methylation, catalyzed by protein arginine methyltransferases (PRMTs), represents a critical post‐translational modification (PTM) regulating processes such as DNA repair, mRNA splicing, cellular signaling.^[^ ^14^ ^]^ PRMTs utilize S‐adenosyl‐methionine (SAM) as a methyl donor to modify substrate arginines.^[^ ^14^ ^]^ The nine mammalian PRMTs are classified into three types: type I (PRMT1–4, 6, 8) enzymes generate aDMA; type II (PRMT5, 7, 9) produce sDMA; and type III (PRMT7 exclusively) catalyzes mono‐methylation (MMA)(14). Dysregulation of PRMTs has been implicated in tumorigenesis, making these enzymes attractive therapeutic targets.^[^ ^14^ ^]^ Notably, while JmjC domain‐containing enzymes demonstrate arginine demethylase activity,^[^ ^15^ ^]^ the existence of dedicated arginine demethylases remains controversial.^[^ ^14^ ^]^

PRMT1, the predominant type I enzyme, contains three conserved domains: 1) an N‐terminal Rossmann‐fold methyltransferase domain harboring the SAM‐binding pocket; 2) a C‐terminal β‐barrel domain forming the substrate‐binding cleft; and 3) an α‐helical dimerization arm mediating oligomerization.^[^ ^16^ ^]^ Its substrates participate in oncogenic processes such as epigenetic regulation, DNA repair, and proliferative signaling.^[^ ^17^ ^]^ Emerging evidence underscores PRMT1 inhibition as a viable therapeutic strategy, with multiple PRMT1 inhibitors demonstrating efficacy against cancer and metabolic disorders in preclinical models.^[^ ^17^ ^]^ However, its clinical utility remains unproven, as no human trials to date have validated the efficacy of PRMT1 inhibition.^[^ ^18^ ^]^ Post‐translational regulation of PRMT1 remains poorly characterized, with established modifications including phosphorylation‐dependent substrate recognition,^[^ ^19^ ^]^ ubiquitination‐involved disruption of protein stability,^[^ ^20^ ^]^ and acetylation‐mediated arrest of PRMT1 in the cytoplasm, which modulates SLC7A11 transcription.^[^ ^21^ ^]^ Other PTMs of PRMT1, such as lactylation, remain largely unexplored.

Protein lactylation, an emerging post‐translational modification (PTM) of lysine residues, has emerged as a key regulatory mechanism in cancer biology.^[^ ^22^ ^]^ This modification influences diverse oncogenic processes, including DNA damage and repair,^[^ ^23^ ^]^ p53‐dependent tumorigenesis,^[^ ^24^ ^]^ mitochondrial metabolic reprogramming,^[^ ^25^ ^]^ and regulated cell death pathways such as ferroptosis and cuproptosis related cell death.^[^ ^26^, ^27^ ^]^ Notably, proteomic studies have identified over 1000 lactylated proteins in multiple malignant cell types,^[^ ^24^, ^28^ ^]^ underscoring its functional relevance to tumor initiation and progression. Our study identifies a hypoxia‐driven post‐translational signaling axis central to metastatic progression, wherein oxygen limitation orchestrates a dynamic enzymatic network governing cytoskeletal remodeling. We demonstrate that hypoxic microenvironments promote the downregulation of HDAC8, the delactylase of PRMT1, thereby promoting hyperlactylation of PRMT1 at conserved lysine residues and allosteric activation of its methyltransferase activity on vimentin at arginine 64 (R64). This PRMT1‐vimentin R64 asymmetric demethylation (aDMA) axis drives cytoskeletal reorganization and cancer cell migratory capacity, with functional validation in xenograft models demonstrating its critical requirement for metastatic colonization. Mechanistically, we characterize KAT7 and KDM4A as antagonistic lactyltransferase and demethylase regulating this node, revealing a hypoxia‐disrupted enzymatic equilibrium. Clinically, vimentin R64 aDMA status correlates significantly with advanced tumor stage and poor survival in triple‐negative breast cancer (TNBC), establishing this modification as a robust prognostic biomarker for metastatic risk. Collectively, our work defines a previously uncharacterized hypoxia‐cytoskeleton regulatory interface with translational relevance for metastatic disease intervention.

## 2 Results

### 2.1 Hypoxia Promotes Vimentin Filament Remodeling and Cancer Cell Migration

**Figure 1:**
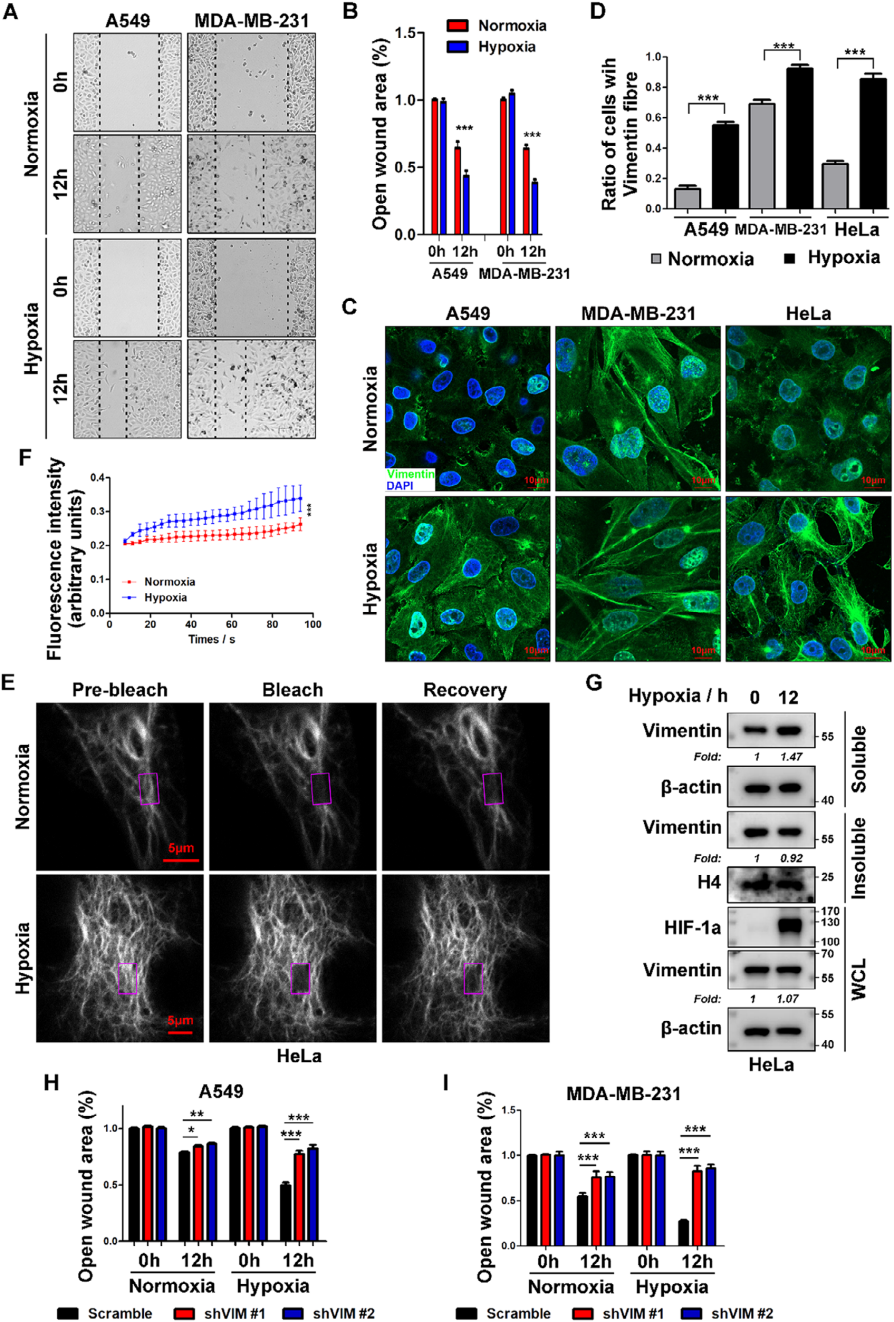
Hypoxia modulates vimentin dynamics and cancer cell migration. A) Hypoxia enhances cell migratory capacity in A549 an MDA‐MB‐231 cells. Wound healing assays were performed under normoxic (21% O_2_) or hypoxic (1% O_2_, 12 h) conditions. Representative images at 0 and 12 h post‐scratch are shown. B) Quantitative analysis of wound closure rates. Relative open wound areas from panel A) were quantified as (final wound area / initial wound area) × 100% using ImageJ (n = 3 independent experiments, 5 fields per well). Cell migration rate was calculated as 100% ‐ relative open wound area, with standardized data processing across replicates. C) Hypoxia induces vimentin filament assembly. Immunofluorescence staining of endogenous vimentin (green) in A549, MDA‐MB‐231, and HeLa cells under normoxic (21% O_2_) or hypoxic (1% O_2_, 12 h) conditions. Nuclei counterstained with DAPI (blue). Confocal microscopy images (scale bar: 10 µm). D) Quantification of filamentous vimentin morphology. Percentage of cells displaying filamentous vimentin was determined by visual scoring of ≥ 50 cells per condition across 3 independent experiments. E) Hypoxia increases vimentin dynamics. FRAP assays in GFP‐vimentin‐expressing HeLa cells: cytoplasmic regions (purple box) were photobleached with a 488‐nm laser, and fluorescence recovery was monitored every 3 s for 100 s after 12 h normoxic/hypoxic treatment. F) Quantitative FRAP analysis. Mobile fraction and recovery time were analyzed by using GraphPad Prism (n = 10 cells per condition, 3 independent experiments). G) Hypoxic stress alters vimentin solubility in HeLa cells. Solubule/insoluble fractionation assay after 12 h normoxia/hypoxia: cell lysates were fractionated into soluble, insoluble, and whole cell lysate (WCL). HIF‐1α served as a hypoxia marker. Western blot analysis probed with anti‐vimentin, with β‐actin (WCL/ soluble fractions) and histone H4 (insoluble fraction) as loading controls. Band intensities normalized to respective controls (n = 3 independent experiments). “Fold” = fold change. H,I) Vimentin depletion attenuates hypoxia‐induced migration. Statistical analysis of wound healing assays from Figure S1E (Supporting Information) (A549) and S1F (MDA‐MB‐231). Relative open wound area was quantified as in (B) (n = 3 independent experiments, 5 fields per well). All experiments performed in ≥3 independent biological replicates unless specified. Quantitative data shown as mean ± SEM with n values indicated. Statistical significance determined by two‐tailed unpaired Student's *t‐*test. ns, *p* > 0.05; *, *p* < 0.05; **, *p* < 0.01; ***, *p* < 0.001.

Solid tumors commonly exhibit hypoxic microenvironments, a recognized driver of metastatic progression.^[^ ^3^, ^4^ ^]^ Notably, lung cancer and breast cancer exhibit particularly aggressive metastatic phenotypes.^[^ ^2^ ^]^ To investigate hypoxia‐induced metastasis mechanisms, we employed wound‐healing assays in two prototypical metastatic models: A549 non‐small cell lung cancer (NSCLC) and MDA‐MB‐231 triple‐negative breast cancer (TNBC) cells. Hypoxic exposure significantly enhanced migratory capacity in both cell lines relative to normoxic conditions (**Figure** **1**A,B). Given vimentin's established role as a critical mediator of metastatic dissemination through EMT(29), and its known structural plasticity during redox perturbation,^[^ ^30^ ^]^ we next characterized its filament assembly under hypoxic conditions. Immunofluorescence (IF) analysis revealed prominent hypoxia‐induced vimentin filament enrichment in both A549 and MDA‐MB‐231 cells (Figure 1C,D), despite unchanged total vimentin protein levels (Figure S1A,B, Supporting Information). To assess the universality of hypoxia‐induced vimentin reorganization, we extended these observations to HeLa cells, a widely utilized cervical cancer cell line and canonical model in cell biology research. In these cells, hypoxic stimulation similarly enhanced filament assembly (Figure 1C,D) while leaving vimentin protein levels unaltered (Figure S1C, Supporting Information), suggesting a conserved regulatory mechanism across epithelial cancer models.

While vimentin filament elongation predominantly occurs via longitudinal annealing of unit‐length filaments through end‐to‐end fusion, emerging evidence supports tetramers subunit exchange with these cytoskeletal structures.^[^ ^31^ ^]^ To investigate whether hypoxic stress influences the subunit exchange rate of vimentin filaments, we performed fluorescence recovery after photobleaching (FRAP) analysis in HeLa cells, selected for their well‐defined vimentin cytoskeletal architecture (Figure 1C). This assay monitored subunit exchange dynamics within vimentin filaments (Figure 1E). Quantitative analysis revealed significantly accelerated fluorescence recovery in hypoxic cells (Figure 1E,F), indicating that hypoxic stress enhances tetramer turnover rates in vimentin filaments. Since vimentin solubility reflects its polymerization status,^[^ ^30^ ^]^ we next conducted biochemical fractionation. Hypoxia induced a 1.47‐fold increase in RIPA‐soluble vimentin, accompanied by a modest 0.08‐fold reduction in RIPA‐insoluble vimentin, while total cellular vimentin levels remained unaltered (Figure 1G). These biochemical findings corroborate the microscopic observations, confirming that hypoxia drives vimentin transition into dynamic filamentous networks.

The observed hypoxia‐dependent reorganization of vimentin prompted us to investigate its functional role in cell migration. Functional validation was assessed using wound‐healing assays in vimentin‐depleted A549 and MDA‐MB‐231 cells (Figure S1D, Supporting Information). While vimentin knockdown reduced baseline migration under normoxia, it completely abrogated hypoxia‐induced motility (Figure 1H,I; Figure S1E,F, Supporting Information), highlighting its critical role in microenvironment‐driven migratory capacity. To assess the pathophysiological relevance, we generated an experimental metastasis model via tail vein injection of LM2 cells with stable shRNA‐mediated vimentin knockdown (Figure S1G,H, Supporting Information). These LM2 cells, a subline established via serial in vivo selection from parental MDA‐MB‐231 breast cancer cells, exhibit significantly augmented metastatic tropism to pulmonary tissues relative to their progenitor counterparts.^[^ ^32^ ^]^ Longitudinal bioluminescence imaging (BLI) monitoring revealed a significant reduction in metastatic colonization following vimentin depletion (Figure S1I,J, Supporting Information). Terminal analyses confirmed marked decreases in both metastatic burden and lesion size in vimentin knockdown groups (Figure S1K,L, Supporting Information), as quantified by quantitative histomorphometric analysis of H&E‐stained sections.

Collectively, these multimodal data demonstrate that hypoxia‐induced tumor cell migration is mechanistically linked to vimentin filament reorganization, with cytoskeletal dynamics potentially serving as a hypoxia‐responsive regulator of experimental metastatic competence.

### 2.2 Vimentin R64 aDMA is Sensitive to Oxygen Deprivation and Critical for Cancer Cell Migration and Experimental Metastasis

**Figure 2:**
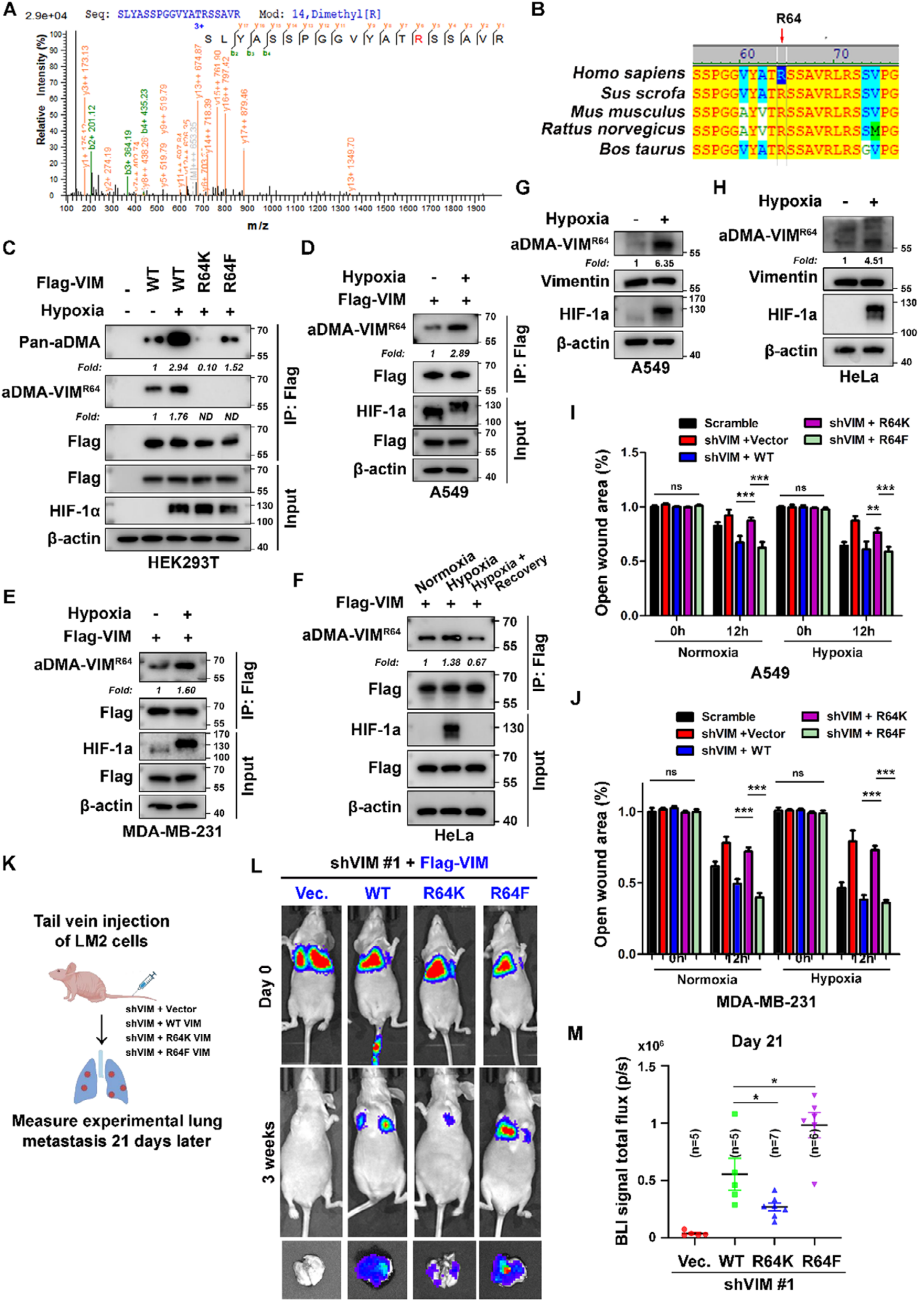
Hypoxia‐driven arginine dimethylation of vimentin at arginine 64 (R64) enhances metastatic potential. A) Identification of R64 dimethylation in vimentin by mass spectrometry (MS). Flag‐vimentin‐expressing HEK293T cells were subjected under hypoxia (1% O_2_, 12 h), followed by immunoprecipitation with anti‐Flag beads, trypsin digestion, and LC‐MS/MS analysis of peptides. B) Evolutionary conservation of vimentin R64. Amino acid alignment around residue 64 of vimentin from Homo sapiens, Sus scrofa, Mus musculus, Rattus norvegicus, and Bos Taurus (Vector NTI software), with the conserved arginine at position 64 highlighted (red arrow). C–F) Immunoprecipitation (IP) analysis of vimentin R64 methylation. C) HEK293T cells expressing Flag‐vimentin WT, R64K, or R64F were treated with normoxia (21% O_2_) or hypoxia (1% O_2_, 12 h). Flag‐vimentin was immunoprecipitated with anti‐Flag beads and probed with pan‐asymmetric dimethylarginine (pan‐aDMA) and aDMA‐VIM^R64^‐specific polyclonal antibodies (custom‐generated using R64 aDMA‐modified peptide as immunogen). D,E) A549 D) and MDA‐MB‐231 E) cells stably expressing Flag‐vimentin were subjected to normoxia/hypoxia followed by IP as in (C). F) Flag‐vimentin‐expressing HeLa cells were treated with normoxia, hypoxia (1% O_2_, 12 h), or hypoxia followed by normoxic recovery (12 h), with R64 methylation detected via aDMA‐VIM^R64^ antibody (n = 3 independent experiments). G,H) Hypoxia increases endogenous vimentin R64 aDMA levels in A549 and HeLa cells. Western blot analysis of endogenous vimentin R64 aDMA in A549 G) and HeLa H) cells under 12 h normoxia/hypoxia, normalized to total vimentin (n = 3 independent experiments). I,J) R64 Adma promotes cell migration under normoxia and hypoxia. Quantitative analysis of wound healing assays from Figure S2D (Supporting Information) (A549, I) and S2E (MDA‐MB‐231, J). Relative open wound area was quantified as (final wound area / initial wound area) × 100% using ImageJ (n = 3 independent experiments, 5 fields per well), with migration rate calculated as 100% ‐ relative open wound area and standardized data processing. K–M) Vimentin R64 aDMA enhances experimental lung metastasis in vivo. K) Schematic of experimental lung metastasis model via tail vein injection with LM2 cells, monitored by weekly bioluminescence imaging (BLI). L) LM2 cells with shVIM‐mediated vimentin knockdown were reconstituted with Flag‐vimentin WT, R64K, or R64F (puromycin/blasticidin selection). BLI quantified metastatic burden at 3 weeks post‐injection when metastatic signals reached plateau. M) Statistical analysis of BLI signals (n values indicated in figure). HIF‐1α served as a hypoxia marker. Western blot band intensities were quantified via ImageJ. Methylated Flag‐vimentin levels in C–F) were normalized to total Flag‐vimentin in IP samples; endogenous methylation in G,H) was normalized to total vimentin. “Fold” = fold change, relative to control. “ND” = not detected. All experiments performed in ≥3 independent biological replicates unless specified. Quantitative data shown as mean ± SD with n values indicated. Statistical significance determined by two‐tailed unpaired Student's *t‐*test. ns, *p* > 0.05; *, *p* < 0.05; **, *p* < 0.01; ***, *p* < 0.001.

While hypoxia promotes vimentin filament assembly and cell migration (Figure 1), vimentin protein levels remained unaltered (Figure S1A–C, Supporting Information). This implies that post‐translational modifications (PTMs) of vimentin likely mediate the hypoxia‐induced enhancement of cell migratory capacity. To further explore this mechanism, we purified exogenous Flag‐vimentin from HEK293T cells exposed to hypoxic stress and performed mass spectrometry (MS) analysis. Strikingly, this analysis identified dimethylation at arginine residue 64 (R64), a residue highly conserved across diverse species (**Figure** **2**A,B). To validate vimentin dimethylation at R64, we generated two site‐specific mutants: arginine 64 was mutated to lysine (R64K) to ablate methylation potential^[^ ^33^ ^]^ and to phenylalanine (R64F) to mimic a constitutively methylated functional state.^[^ ^34^ ^]^ Immunoblot analysis of immunoprecipitates with a pan‐aDMA antibody revealed robust hypoxia‐induced asymmetric dimethylation (aDMA) of vimentin (Figure 2C). Notably, both Flag‐tagged vimentin R64K and R64F mutants exhibited substantial reductions in aDMA levels under hypoxic conditions, though residual aDMA signals remained (Figure 2C). These findings suggest that while R64 represents the primary hypoxic‐responsive methylation site in vimentin, additional arginine residues may undergo aDMA under hypoxic conditions. These findings underscored the limitations of pan‐aDMA antibodies for site‐specific detection, thereby prompting the development a novel, custom‐designed antibody specific for vimentin asymmetric dimethylation at R64 (aDMA‐vimentin^R64^, hereafter referred to as aDMA‐VIM^R64^). Upon receiving the purified antibody, which had undergone initial specificity test via an ELISA‐based modified peptide competition assay, we conducted a series of mutants‐based specificity assays to further validate its specificity for both endogenous and exogenous vimentin R64 aDMA (Figure S2A–D, Supporting Information). Western blot analysis showed that depletion of endogenous vimentin abolished both vimentin and the aDMA‐VIM^R64^ signal (Figure S2A, Supporting Information). R64 methylation of exogenously expressed vimentin was exclusively detected in wild‐type (WT) Flag‐tagged vimentin samples, but not in the R64K or R64F mutants, in both input lysates and immunoprecipitated fractions (Figure S2B, Supporting Information). In contrast, whole‐cell lysates (WCL) from cells expressing Flag‐tagged vimentin WT, R64K, R64F or empty vector controls exhibited comparable detectable levels of total Flag‐vimentin protein (Figure S2B, Supporting Information). Collectively, these results demonstrate that the aDMA‐VIM^R64^ antibody displays high specificity for vimentin R64 aDMA in Western blot analyses. To further validate the antibody's specificity, we utilized RKO cells, which lack vimentin expression (Figure S2C, Supporting Information). Western blot analysis revealed that exogenous Flag‐tagged vimentin R64 aDMA signals were exclusively detected in wild‐type (WT) Flag‐vimentin‐expressing samples, with no signal detected in R64K or R64F mutant lysates (Figure S2D, Supporting Information). These results further confirm that the aDMA‐VIM^R64^ antibody exhibits robust specificity for vimentin R64 aDMA in both endogenous and heterologous contexts. Using the aDMA‐VIM^R64^ antibody, we observed that hypoxic stimulation induced vimentin R64 aDMA in HEK293T cells (Figure 2C). While this site‐specific antibody detected a modest 1.76‐fold induction – a magnitude less pronounced that the global pan‐aDMA signal detected by the pan‐aDMA antibody – the latter revealed a 2.94‐fold induction under identical experimental conditions (Figure 2C). This discrepancy highlights R64 as a major hypoxia‐responsive methylation site while suggesting additional contributions from other modified arginine residues. To determine whether this response is cell‐type‐specific, we examined vimentin R64 aDMA in A549, MDA‐MB‐231, and HeLa cells. While the magnitude of induction varied, all cell lines exhibited a consistent upward trend in vimentin R64 aDMA under hypoxic conditions (Figure 2D–F). Additionally, we analyzed endogenous vimentin R64 aDMA and observed comparable hypoxia‐driven increases in A549 and HeLa cells (Figure 2G,H), demonstrating that this modification represents a conserved response across diverse cell types. To characterize the dynamics of vimentin R64 aDMA, we subjected HeLa cells stably expressing Flag‐vimentin to hypoxia followed by normoxic recovery. Vimentin R64 aDMA levels significantly decreased upon reoxygenation (Figure 2F), indicating reversibility of this modification in response to hypoxia‐reoxygenation stress. Using cobalt chloride (CoCl_2_) to mimic hypoxic stress,^[^ ^35^ ^]^ we performed time‐course analyses and observed a sustained increase in endogenous vimentin R64 aDMA within 24 h of treatment (Figure S2E, Supporting Information). Collectively, these data demonstrate that while baseline R64 aDMA exists under normoxia, hypoxic stress induces a dynamic, reversible elevation of this aDMA modification, underscoring its role in adaptive cellular responses.

To investigate the role of vimentin R64 aDMA in hypoxia‐induced cancer cell migration, we generated vimentin‐knockdown A549 and MDA‐MB‐231 cell lines and re‐expressed exogenous wild‐type (WT) vimentin or R64‐mutated variants (R64K and R64F) (Figure S2F, Supporting Information). Wound‐healing assays revealed that vimentin depletion significantly impaired migration in both cell lines under normoxic and hypoxic conditions. Re‐expression of WT or R64F vimentin (but not R64K) rescued these migratory defects (Figure 2I,J; Figure S2G,H, Supporting Information), indicating that R64 aDMA is critical for vimentin‐dependent cell migration. To assess metastatic potential in vivo, we employed a mouse xenograft metastasis model using LM2 cells with vimentin knockdown or re‐expression of WT, R64K, or R64F Flag‐tagged vimentin (Figure 2K; Figure S2I, Supporting Information). BLI analysis of live animals and isolated lung tissues demonstrated that re‐expression of wild‐type (WT) vimentin or its R64F mutant, but not the R64K mutant, rescued the experimental metastatic potential of vimentin‐depleted LM2 cells (Figure 2L,M). H&E staining of lung sections confirmed this phenotype, with WT and R64F re‐expression leading to more numerous and larger experimental metastatic nodules, whereas R64K re‐expression resulted in minimal and smaller lesions (Figure S2J,K, Supporting Information). These data establish R64 aDMA as a critical determinant of experimental metastasis in vivo. Collectively, our findings demonstrate that vimentin R64 aDMA is essential for cell migration under both normoxia and hypoxia, and experimental metastatic progression, highlighting its central role in tumor cell dissemination.

### 2.3 Vimentin R64 aDMA Correlates with Poor Clinical Outcomes in TNBC

To evaluate the translational relevance of our findings, we first validated the aDMA‐VIM^R64^ antibody for immunohistochemical (IHC) applications using cancer cell lines (Figure S3A–C, Supporting Information). Consistent with Western blot (WB) analyses (Figure 2E,F,H), hypoxic treatment enhanced aDMA‐VIM^R64^ signals in both MDA‐MB‐231 and HeLa cells (Figure S3A,B, Supporting Information), whereas vimentin knockdown nearly eliminated these signals under both normoxic and hypoxic conditions (Figures S1D and S3A, Supporting Information). Furthermore, robust antibody signals were exclusively detected in RKO cells (a vimentin‐deficient cell line) expressing wild‐type Flag‐tagged vimentin, while only faint non‐specific staining was observed in RKO cells transfected with empty vector or R64 variant constructs (Figures S2D and S3C, Supporting Information). Together, these data confirm that the aDMA‐VIM^R64^ antibody is suitable for IHC applications.

**Figure 3:**
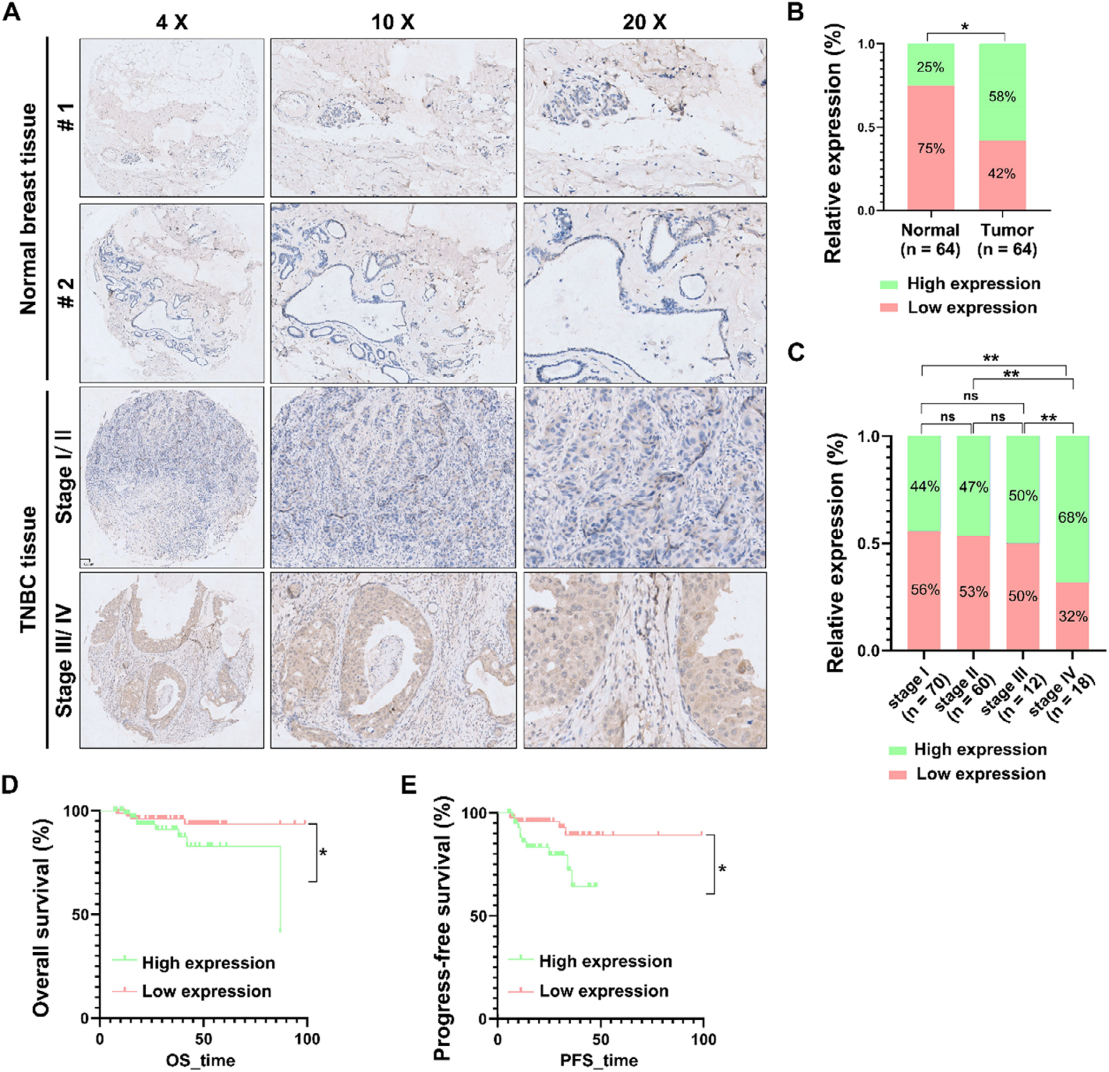
Clinical significance of vimentin R64 aDMA in triple‐negative breast cancer (TNBC) progression. A) Immunohistochemical (IHC) analysis of aDMA‐vimentin^R64^ levels in normal tissues and TNBC clinical specimens across different stages. Tissue microarrays were stained with aDMA‐VIM^R64^ antibody, counterstained with hematoxylin, and imaged at 4x, 10x, and 20x magnification. B) Quantitative assessment of IHC staining intensity for aDMA‐VIM^R64^ in TNBC tumors (n = 64) and adjacent non‐cancerous tissues (n = 64). Semi‐quantitative H‐scores (0‐300) were independent assigned by two pathologists, calculated as (percentage of positive cells × intensity score: 0 = negative, 1 = weak, 2 = moderate, 3 = strong). C) Comparison of aDMA‐VIM^R64^ H‐scores across TNBC tumors stratified by histological grade (Grade I, n = 70; Grade II, n = 60; Grade III, n = 12; Grade IV, n = 18). D,E) Kaplan‐Meier survival curves correlating aDMA‐VIM^R64^ levels with clinical outcomes in TNBC patients. Patients were divided into high/low expression groups based on median H‐score. D) Overall survival (OS) and E) progression‐free survival (PFS) were calculated from surgery date to death or disease progression, respectively. Quantitative data in B–E) are shown as mean ± SD with n values indicated. Statistical analysis: two‐tailed unpaired Student's *t* test for B,C); two‐sided log‐rank test for D) and E). ns, *p* > 0.05; *, *p* < 0.05; **, *p* < 0.01.

Subsequently, we systematically assessed R64‐methylated vimentin levels in clinical TNBC specimens using tumor tissue microarray (TMA) technology. IHC staining and intensity analysis revealed significantly elevated aDMA‐VIM^R64^ signals in TNBC tissues compared to adjacent non‐tumorous breast epithelium (**Figure** **3**A). Quantitative analysis confirmed a marked enrichment of vimentin R64 aDMA in malignant tissues (Figure 3B), with particularly elevated expression observed in advanced‐stage tumors (Figure 3A,C). Kaplan‐Meier survival analysis demonstrated that high aDMA‐VIM^R64^ levels were strongly associated with poor overall survival (OS) and progression‐free survival (PFS) in TNBC patients (Figure 3D,E). Collectively, these findings establish increased aDMA‐VIM^R64^ levels as a stage‐dependent hallmark of TNBC progression and a prognostic biomarker for poor clinical outcomes.

In a representative cohort of patients with invasive TNBC, aDMA‐VIM^R64^ levels were evaluated in metastatic contexts. IHC analysis revealed significantly higher aDMA‐VIM^R64^ signal intensity in stage IV lesions compared to stage II counterparts (Figure S3D–F, Supporting Information), indicating a potential association between vimentin R64 aDMA and metastatic progression.

### 2.4 Vimentin R64 aDMA is Critical for Hypoxia‐Driven Cytoskeletal Remodeling

**Figure 4:**
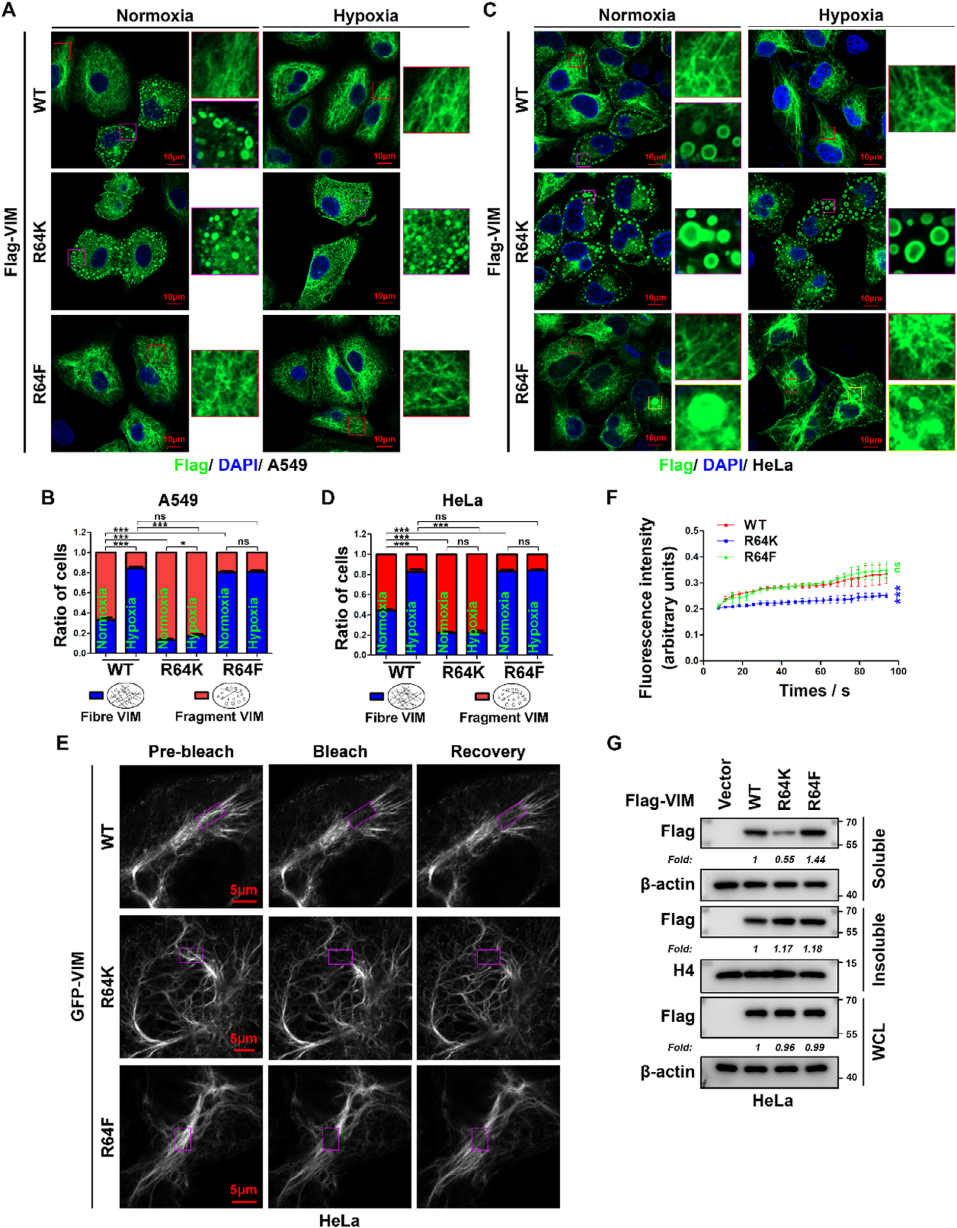
Vimentin R64 methylation promotes filament assembly and dynamics in cancer cells. A,C) R64 aDMA enhances vimentin filamentous network formation. A549 A) and HeLa C) cells transfected with Flag‐vimentin WT, R64K, or R64F were subjected to normoxia (21% O_2_) or hypoxia (1% O_2_, 12 h), followed by immunofluorescence staining with anti‐Flag antibody (green) and DAPI (blue) counterstaining. Confocal microscopy images show representative vimentin morphology in indicated genotype; red/purple boxes indicate magnified regions (scale bar: 10 µm; n = 3 independent experiments). B,D) Quantitative analysis of vimentin morphology. Blind scoring of ≥50 cells per condition across 3 independent experiments categorized cells into filamentous (long fibers) or fragmented (diffuse/short oligomers) vimentin populations in A) and C). E) R64 aDMA is essential for hypoxic vimentin dynamics. FRAP analysis in GFP‐vimentin WT/R64K/R64F‐expressing HeLa cells: cytoplasmic regions (purple box) were photobleached with a 488‐nm laser after 12 h hypoxia, and fluorescence recovery was monitored every 3 s for 100 s. F) FRAP quantification. Mobile fraction and recovery time were analyzed using GraphPad Prism (mean ± SEM, n = 10 cells per condition, 3 independent experiments; two‐tailed unpaired Student's *t‐*test. ns, *p* > 0.05; *, *p* < 0.05; ***, *p* < 0.001). G) R64 aDMA regulates vimentin solubility under hypoxia. HeLa cells expressing Flag‐vimentin variants (WT/R64K/R64F) were fractionated into soluble, insoluble, and whole cell lysate (WCL) after 12 h hypoxia. Western blots probed with anti‐Flag antibody, anti‐β‐actin (WCL/soluble control), and anti‐histone H4 (insoluble control). Band intensities were normalized to loading controls and expressed as fold change (“Fold”) relative to WT (n = 3 independent experiments).

To investigate the functional role of vimentin R64 aDMA in hypoxia‐driven cytoskeletal remodeling, we transfected wild‐type (WT) vimentin or R64‐site mutants (R64K and R64F) into A549 and HeLa cells. Immunofluorescence (IF) imaging revealed that hypoxic stimulation induced robust assembly of filamentous vimentin networks in wild‐type (WT) vimentin‐transfected cells (**Figure** **4**A–D). In contrast, the R64K mutation disrupted filament assembly under both normoxia and hypoxia. Notably, the R64F mutation preserved highly organized filamentous structures under both normoxic and hypoxic conditions (Figure 4A–D). Intriguingly, ≈5% of HeLa cells expressing the R64F mutant exhibited larger cytoplasmic puncta with solid circular morphology (Figure 4C), a phenotype distinct from the fragmented filaments (hollow circular structures) observed in WT and R64K‐expressing cells (Figure 4C). The biological significance of these aggregates, which may be associated with the formation of hyper‐stable filaments, warrants further investigation.

To characterize the dynamics of vimentin filaments, we conducted live‐cell FRAP assays in HeLa cells stably expressing GFP‐tagged WT vimentin or its mutants (R64K and R64F) following hypoxia treatment. FRAP analysis revealed rapid fluorescence recovery within 100 s post‐bleach in wild‐type (WT) and R64F‐expressing cells, reflecting active filament subunit turnover (Figure 4E,F). In contrast, R64K‐expressing cells exhibited negligible fluorescence recovery over the same time period, suggesting stable, non‐dynamic filament networks (Figure 4E,F). Biochemical fractionation assays validated these observations: R64K mutants showed a 0.45‐fold reduction in RIPA‐soluble vimentin (0.55‐fold relative to WT controls), whereas R64F mutants exhibited a 1.44‐fold increase in solubility (Figure 4G). Total exogenous vimentin levels remained unchanged in hypoxic HeLa cells across all groups (Figure 4G). Collectively, these data establish R64 aDMA as a critical regulatory switch governing vimentin filament dynamics and turnover during hypoxic stress.

### 2.5 PRMT1‐Mediated Vimentin R64 Methylation Drives Cytoskeletal Remodeling

**Figure 5:**
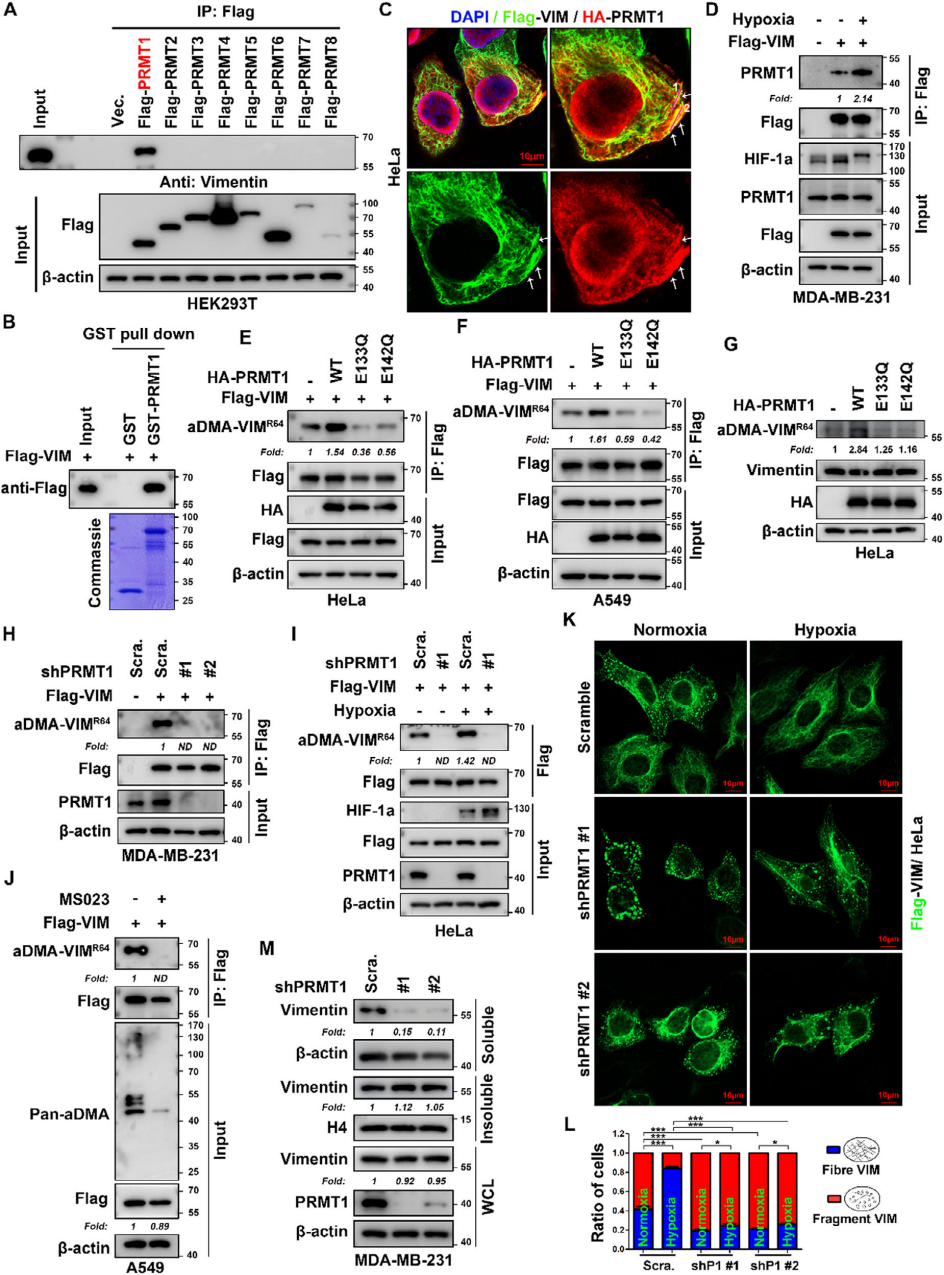
PRMT1 mediates vimentin R64 asymmetric dimethylation and regulates filament assembly. A) Selective interaction between vimentin and Flag‐PRMT1 in HEK293T cells. Cells were transfected with Flag‐PRMT1‐8 lentiviruses, and 48 h post‐transfection, lysates were immunoprecipitated with anti‐Flag beads. Blots were probed with anti‐vimentin (endogenous) and anti‐Flag (PRMT family members). B) GST pull‐down confirms physical interaction between PRMT1 and vimentin. Recombinant GST‐PRMT1 (v5 transcript) or GST control was expressed in *E.coli*, purified via glutathione agarose beads, and incubated with Flag‐vimentin‐transfected HEK293T cell lysates. Bound proteins were detected by Western blot with anti‐Flag antibody (n = 3 independent experiments). C) Co‐localization of PRMT1 and vimentin in HeLa cells. Cells co‐transduced with Flag‐vimentin and HA‐PRMT1 (v5 transcript) were stained with anti‐Flag (green) and anti‐HA (red) antibodies, with DAPI (blue) counterstaining. White arrows indicate cytoplasmic co‐localization. D) Hypoxia enhances PRMT1‐vimentin interaction in MDA‐MB‐231 cells. Cells stably expressing Flag‐vimentin were treated with normoxia/hypoxia (1% O_2_, 12 h), followed by co‐IP with anti‐Flag beads. Endogenous PRMT1 was detected with anti‐PRMT1 antibody and normalized to Flag‐vimentin in IP samples (n = 3 independent experiments). E,F) PRMT1 overexpression promotes vimentin R64 aDMA in HeLa and A549 cells. Cells expressing Flag‐vimentin were transfected with HA‐PRMT1 WT or enzymatical mutants (E133Q, E142Q). Flag‐vimentin were immunoprecipitated, and R64 aDMA was detected with aDMA‐VIM^R64^ antibody, normalized to total Flag‐vimentin in IP samples (n = 3 independent experiments). G) PRMT1 overexpression increases endogenous vimentin R64 methylation in HeLa cells. Cells transfected with HA‐PRMT1 variants were lysed, and endogenous R64 aDMA was probed with aDMA‐VIM^R64^ antibody, normalized to total vimentin (n = 3 independent experiments). H,I) PRMT1 knockdown reduces vimentin R64 aDMA in MDA‐MB‐231 and HeLa cells. Cells expressing Flag‐vimentin were transduced with shPRMT1 or shScramble lentivirus, treated with normoxia/hypoxia for 12 h, and subjected to IP as in (E) (n = 3 independent experiments). J) Pharmacological inhibition of PRMT1 blocks vimentin R64 aDMA. A549 cells expressing Flag‐vimentin were treated with 15uM MS023 (type I PRMT inhibitor) or DMSO for 24 h, followed by IP and aDMA‐VIM^R64^ detection. Pan‐aDMA antibody validated global aDMA changes (n = 3 independent experiments). K,L) PRMT1 depletion disrupts vimentin filamentous network formation. K) HeLa cells with Flag‐vimentin were transfected with shPRMT1 (#1, #2) or shScramble, selected with puromycin, and stained with anti‐Flag (green) antibody after normoxia/hypoxia treatment for 12 h. L) Quantitative morphology analysis: blind scoring of ≥50 cells per condition categorized filamentous versus fragmented vimentin (mean ± SEM, n = 3 independent experiments; two‐tailed unpaired Student's *t‐*test. *, *p* < 0.05; ***, *p* < 0.001). M) PRMT1 knockdown alters vimentin solubility in MDA‐MB‐231 cells. Cells were fractionated into soluble, insoluble, and whole cell lysate (WCL) fractions, probed with anti‐vimentin, and normalized to β‐actin (soluble/WCL) or histone H4 (insoluble). Band intensities were quantified as fold change relative to shScramble (n = 3 independent experiments). HIF‐1α served as a hypoxia marker. All experiments performed in ≥3 independent biological replicates unless specified. “Fold”, fold change; “ND”, not detected.

To elucidate the molecular basis of vimentin methylation, we transfected HEK293T cells with vectors encoding protein arginine methyltransferases (PRMTs) and performed co‐immunoprecipitation (co‐IP) assays. Western blot analysis revealed that endogenous vimentin specifically co‐precipitated with Flag‐PRMT1 when Flag‐PRMT1 was immunoprecipitated using an anti‐Flag beads (**Figure** **5**A). Reciprocal co‐IP experiments using anti‐Flag beads to immunoprecipitate Flag‐vimentin demonstrated that HA‐PRMT1 co‐precipitated with Flag‐vimentin (Figure S4A, Supporting Information), confirming the bidirectional interaction. GST pull‐down analysis further demonstrated a direct physical interaction between PRMT1 with vimentin (Figure 5B), while IF analysis revealed their robust cytoplasmic co‐localization (Figure 5C; Figure S4B, Supporting Information). To investigate how this interaction dynamics under hypoxic stress, we employed CoCl_2_ treatment to mimic temporal hypoxia over time. Co‐IP analysis revealed a biphasic response: an initial decrease in PRMT1‐vimentin binding at 2 h, followed by a significant increase at 8 h in HEK293T cells (Figure S4C, Supporting Information). Prolonged hypoxic treatment (12 h) enhanced this interaction in MDA‐MB‐231 and HeLa cells (Figure 5D; Figure S4D, Supporting Information), indicating that hypoxia promotes the association between PRMT1 and vimentin.

Building on these confirmed interaction, we performed deletion mapping to identify the binding domains. Deletion mapping identified the vimentin‐binding domain of PRMT1 to residues 25‐343 (Figure S4E,F, Supporting Information). Truncation of the N‐terminal 1‐24 amino acids (aa) retained binding to Flag‐vimentin in co‐IP assays, whereas deletion of the 25‐343 aa segment completely abolished the interaction. Notably, truncations removing either the 1‐165 aa or 166‐343 aa subregions, which disrupt the integrity of the 25‐343 region, also failed to bind vimentin, indicating that the intact 25‐343 aa domain is essential for interaction (Figure S4E,F, Supporting Information). Further truncation studies will be needed to determine whether this domain can be narrowed to smaller subregions, such as 25‐165 aa. Conversely, when assessing vimentin's binding to PRMT1, all truncated vimentin mutants retained some levels of interaction, though constructs lacking residues 264‐283 or 294‐405 exhibited reduced binding affinity in co‐IP assays (Figure S4G,H, Supporting Information). These results indicate that while these regions contribute to the interaction, they are not strictly essential for PRMT1‐vimentin binding.

Biochemical evidence of the PRMT1‐vimentin interaction suggests PRMT1 may regulate vimentin arginine methylation. Immunoblot analysis using pan‐aDMA antibodies revealed a marked increase in asymmetric dimethylarginine (aDMA) levels in Flag‐vimentin from HEK293T cells overexpressing wild‐type (WT) PRMT1 ‐ but not in cells expressing catalytically inactive PRMT1 mutants (E133Q and E142Q) (Figure S4I, Supporting Information). Site‐specific immunodetection using the aDMA‐VIM^R64^ antibody demonstrated a significant 1.34‐fold increase in vimentin R64 aDMA following WT PRMT1 overexpression (Figure S4J, Supporting Information), though the magnitude of R64 methylation enhancement was less pronounced than the global aDMA signal detected by pan‐aDMA antibodies (Figure S4I,J, Supporting Information). These findings establish PRMT1 as a bona fide methyltransferase for vimentin methylation, with R64 serving as a potentially predominant, though not exclusive, target site. In cancer cell lines, overexpression of wild‐type (WT) PRMT1, but not catalytically inactive mutants (E133Q and E142Q), enhanced vimentin R64 aDMA in both HeLa and A549 cells stably expressing Flag‐vimentin (Figure 5E,F). Similar effects were observed on endogenous vimentin R64 aDMA (Figure 5G). Furthermore, knockdown of endogenous PRMT1 followed by reintroduction of wild‐type (WT) PRMT1, but not catalytically inactive mutant (E133Q/ E142Q, 2EQ), significantly restored vimentin R64 aDMA (Figure S4K, Supporting Information). This confirms the conserved role for PRMT1 in mediating vimentin R64 aDMA across different cell lines. In contrast, PRMT1 depletion almost completely abrogated vimentin R64 aDMA in MDA‐MB‐231 cells (normoxia) and HeLa cells (normoxia/hypoxia) (Figure 5H,I), indicating that PRMT1 is essential for vimentin R64 aDMA. MS023, a PRMT type I inhibitor (which targets PRMT1, 3, 4, 6 and 8), is commonly recognized as a PRMT1 inhibitor in translational research.^[^ ^36^ ^]^ Consistent with this, MS023 treatment for 24 h abolished vimentin R64 aDMA in both A549 and HeLa cells (Figure 5J; Figure S4L, Supporting Information). Collectively, these findings establish PRMT1 as the primary methyltransferase responsible for vimentin dimethylation.

To elucidate PRMT1's role in vimentin filament assembly, IF analysis revealed that PRMT1 depletion led to accumulation of fragmented vimentin networks under both normoxic and hypoxic conditions (Figure 5K,L). While hypoxia modestly increased filamentous vimentin in PRMT1‐depleted cells, the majority still exhibited fragmented morphology (Figure 5K,L). Soluble fractionation assays confirmed these findings, showing significantly reduced vimentin levels in the RIPA‐soluble fraction of PRMT1‐knockdown MDA‐MB‐231 cells, despite minimal changes in total cellular vimentin (Figure 5M). Together, these findings indicate that PRMT1 is critical for maintaining vimentin solubility and promoting filament assembly.

### 2.6 KDM4A is the Demethylase for Vimentin R64 aDMA

Given the absence of a definitively identified dedicated arginine demethylase, emerging evidence suggests that certain lysine demethylases (KDMs) may exhibit promiscuous activity toward arginine residues.^[^ ^37^ ^]^ To identify potential demethylases for vimentin, we conducted a small‐scale shRNA screen in stable Flag‐vimentin‐expressing HeLa cells, depleting candidate KDM family members followed by IP assays. Importantly, KDM4A depletion significantly increased vimentin R64 aDMA (Figure S5A, Supporting Information). To further validate the generality of this phenotype, we knocked down KDM4A in MDA‐MB‐231, A549, and HEK293T cells and assessed Flag‐tagged vimentin R64 aDMA. Strikingly, KDM4A depletion consistently induced a similar increase in aDMA levels across all cell lines (Figure S5B–D, Supporting Information), with comparable elevations observed in endogenous vimentin R64 aDMA in HeLa cells (Figure S5E, Supporting Information). Notably, co‐IP assays in HEK293T cells revealed a physical interaction between KDM4A and vimentin, which was abolished upon KDM4A depletion (Figure S5F, Supporting Information). Conversely, overexpression of wild‐type (WT) KDM4A, but not its catalytically inactive H188A mutant, reduced vimentin R64 aDMA in multiple cell lines (Figure S5G–J, Supporting Information). Collectively, these findings establish KDM4A as the demethylase for vimentin hypomethylation at the R64 residue.

### 2.7 Hypoxia‐Induced PRMT1 lactylation at K134/ K145 Drives Vimentin Methylation and Cytoskeletal Remodeling

**Figure 6:**
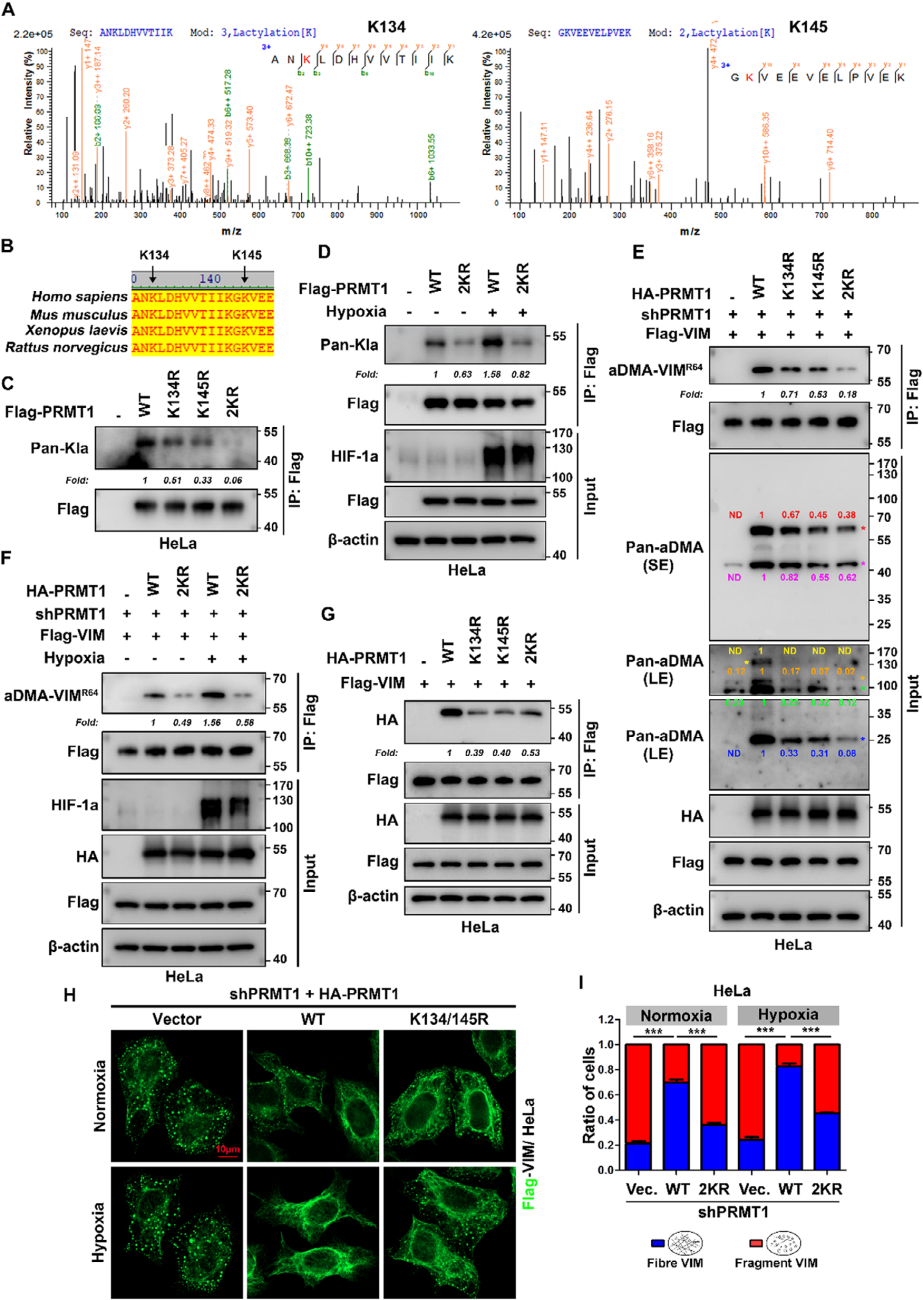
Hypoxia‐induced lactylation of PRMT1 at K134/K145 enhances its methyltransferase activity toward vimentin. A) Identification of K134 and K145 lactylation sites by mass spectrometry (MS). HEK293T cells transfected with Flag‐PRMT1 (v2 transcript) were subjected to hypoxia (1% O_2_) for 12 h, followed by immunoprecipitation and LC‐MS/MS analysis of trypsin‐digested peptides. B) Evolutionary conservation of PRMT1 K134/K145. Amino acid alignment of PRMT1 (v2 transcript) surrounding K134/K145 across Homo sapiens, Mus musculus, Xenopus laevis, and Rattus norvegicus (Vector NTI software), with conserved lysine residues 134 and 145 highlighted (black arrows). C) Site‐specific lactylation analysis of PRMT1 mutants. HeLa cells transfected with Flag‐PRMT1 WT, K134R, K145R, or 2KR (K134R/K145R) lentivirus were subjected to immunoprecipitation with anti‐Flag beads 48 h post‐transfection, followed by Western blot analysis with pan‐lactyllysine (pan‐Kla) antibody (n = 3 independent experiments). D) Hypoxia promotes PRMT1 lactylation at K134/K145. HeLa cells expressing Flag‐PRMT1 WT or 2KR (K134R/K145R) were treated with normoxia/hypoxia (1% O_2_, 12 h), and lactylation was detected via anti‐Flag IP and pan‐Kla antibody (n = 3 independent experiments). E,F) Functional rescue of vimentin methylation by PRMT1 lactylation mutants. HeLa cells with PRMT1 knockdown were reconstituted with HA‐PRMT1 WT, K134R, K145R, or 2KR (K134R/K145R) via lentivirus transfection, along with Flag‐vimentin transfection. E) normoxic conditions; F) normoxia/hypoxia (1% O_2_, 12 h) treatment. Flag‐vimentin was immunoprecipitated, and R64 aDMA was detected with aDMA‐VIM^R64^ antibody, normalized to total Flag‐vimentin in IP samples (n = 3 independent experiments). G) PRMT1 lactylation mutants exhibit weakened interaction with vimentin. HeLa cells expressing Flag‐vimentin were transfected with HA‐PRMT1 variants, followed by co‐IP with anti‐Flag beads and detection of bound HA‐PRMT1 (n = 3 independent experiments). H,I) K134/K145 lactylation is required for vimentin filament maintenance. H) HeLa cells with PRMT1 knockdown were rescued with WT or 2KR (K134R/K145R) PRMT1, treated with normoxia/hypoxia (1% O_2_, 12 h), and stained anti‐Flag (green) for Flag‐vimentin detection. I) Quantitative morphology analysis: blind scoring of ≥50 cells per condition categorized filamentous versus fragmented vimentin (mean ± SEM, n = 3 independent experiments; two‐tailed unpaired Student's *t‐*test. ***, *p* < 0.001). HIF‐1α served as a hypoxia marker. Lactylated Flag‐PRMT1 levels in C–E) were normalized to total Flag‐PRMT1; methylated Flag‐vimentin in E,F) was normalized to total Flag‐vimentin; HA‐PRMT1 in G) was normalized to Flag‐vimentin in IP samples. Pan‐aDMA signals (indicated with colored stars) in E) were normalized to β‐actin. Fold change relative to control is indicated where applicable as “Fold” (“ND”= not detected). All experiments performed in ≥3 independent biological replicates unless specified.

To uncover the molecular mechanism by which PRMT1 drives vimentin R64 aDMA during hypoxia, we conducted mass spectrometry (MS) on purified Flag‐PRMT1 from HEK293T cells treated with normoxia and hypoxia. This analysis identified two evolutionarily conserved lysine residues ‐ K134 and K145 – within PRMT1's catalytic domain, both of which showed robust enrichment of lactylation under hypoxia (**Figure** **6**A,B; Figure S6A, Supporting Information). Subsequent IP assays using a pan‐lysine lactylation (Kla) antibody(38) revealed that single (K134R or K145R) and double (K134R/K145R) mutations reduced PRMT1 lactylation, with the double mutant almost completely eliminating this modification (Figure 6C). Notably, hypoxic stress induced a 1.58‐fold increase in lactylation of wild‐type (WT) PRMT1, whereas the K134R/K145R (2KR) mutant showed no such response (Figure 6D). These results establish hypoxia as a key inducer of PRMT1 lactylation at the K134 and K145 sites.

To assess the functional role of PRMT1 lactylation in vimentin R64 aDMA, we overexpressed wild‐type (WT) PRMT1 or its lactylation‐deficient mutants (K134R, K145R, or K134R/K145R) in HeLa cells. Western blot analysis revealed that WT PRMT1 significantly enhanced vimentin R64 aDMA, whereas single (K134R and K145R) mutants moderately attenuated this effect, and the double (K134R/K145R) mutant nearly abolished it (Figure S6B, Supporting Information). To further validate this, we performed rescue experiments in cell lines stably expressing Flag‐vimentin with endogenous PRMT1 knocked down. WT PRMT1 fully restored vimentin R64 aDMA, while single mutants (K134R or K145R) reduced recovery by 29% and 47%, respectively. The double mutant (K134R/K145R, 2KR) achieved only 18% restoration compared to WT (Figure 6E). Notably, the K134R/K145R double mutation nearly abolished hypoxia‐induced vimentin R64 aDMA in WT PRMT1 re‐expressed HeLa cells (Figure 6F). Collectively, these data indicate that K134/K145 lactylation is critical for PRMT1‐mediated vimentin R64 aDMA under both normoxia and hypoxia.

Building on these functional insights, we sought to determine whether PRMT1 lactylation influences substrate specificity. Global aDMA profiling revealed that wild‐type (WT) PRMT1 expression enhanced methylation of diverse substrates, an effect significantly attenuated by lactylation‐deficient mutants (K134R, K145R, K134R/K145R) (Figure S6B, Supporting Information). In PRMT1‐depleted HeLa cells, WT PRMT1 re‐expression fully restored global aDMA levels, while single mutants (K134R and K145R) showed partial recovery and the double mutant (K134R/K145R) exhibited negligible restoration (Figure 6E). These findings establish K134/K145 lactylation as indispensable for maintaining PRMT1's enzymatic activity toward both vimentin and its broader substrates. To elucidate the structural basis of lactylation‐mediated regulation, we mapped the spatial relationship between K134/K145 and PRMT1's conserved methyltransferase motifs. Both residues reside within the catalytic domain (aa 50‐193) but are distal to the six canonical motifs essential for methyltransferase activity,^[^ ^16^, ^39^ ^]^ positioned between the post‐I and motif II regions (Figure S6A, Supporting Information). This topological arrangement suggests that lactylation at K134/K145 modulates PRMT1 activity through non‐canonical mechanisms, distinct from direct perturbation of catalytic motifs. Molecular docking and structural modeling predicted distinct regulatory roles for each residue. K134 is situated within a conserved flexible loop (residues 133–139) (Figure S6C‐C’, Supporting Information), where lactylation may induce conformational changes that propagate to the active site (Figure S6D‐D’, Supporting Information). In contrast, K145 localizes to the entrance of a conserved substrate channel (Figure S6E, Supporting Information), and lactylated at K145 (lac‐K145) is predicted to sterically restrict substrate access to this channel (Figure S6F, Supporting Information). Collectively, these structural insights propose a dual regulatory mechanism for PRMT1 lactylation: (i) allosteric modulation of active site dynamics via K134, and (ii) direct regulation of substrate accessibility via K145.

To investigate whether lactylation influences the subcellular distribution of PRMT1, we performed IF analysis in HeLa cells under normoxic and hypoxic conditions. Staining for Flag‐tagged PRMT1 variants revealed consistent subcellular localization patterns, with fewer than 20% of cells exhibiting dominant nuclear localization (Figure S7A, Supporting Information) and over 80% showing predominant cytoplasmic distribution (Figure S7B, Supporting Information). Quantitative analysis of nucleus‐to‐cytoplasm ratios confirmed no statistically significant differences across PRMT1 variants (Figure S7C, Supporting Information), indicating that lactylation‐deficient mutations (K134R, K145R, K134R/K145R) do not alter PRMT1's subcellular distribution. Notably, cytoplasmic PRMT1 variants displayed differential co‐localization with endogenous vimentin (Figure S7B,D, Supporting Information). Hypoxia enhanced co‐localization between wild‐type (WT) Flag‐PRMT1 and endogenous vimentin, an effect blunted by lactylation‐deficient mutations (Figure S7B,D, Supporting Information). Specifically, lactylation‐deficient mutants (K134R, K145R, K134R/K145R) exhibited reduced vimentin co‐localization under normoxia (Figure S7B,D, Supporting Information), mirroring the reduction in PRMT1‐vimentin binding observed in co‐IP assays (Figure 6G). These complementary findings mechanistically link K134/K145 lactylation to strengthened PRMT1‐vimentin interactions, consistent with prior observations that hypoxia promotes both K134/K145 lactylation (Figure 6D) and PRMT1‐vimentin association (Figure 5D; Figure S4D, Supporting Information). IF‐based functional analysis in PRMT1‐knockdown HeLa cells revealed that WT PRMT1 re‐expression restored filamentous vimentin networks, whereas the K134R/K145R double mutant failed to rescue this phenotype under both normoxia and hypoxia (Figure 6H,I). This indicates that PRMT1 lactylation at K134/K145 is critical for vimentin cytoskeletal remodeling. Further validation via wound healing assays in PRMT1‐depleted MDA‐MB‐231 and A549 cells showed that WT PRMT1 re‐expression rescued migratory defects under normoxia and hypoxia, while lactylation‐deficient mutants did not (Figure S8A–F, Supporting Information). Collectively, these findings establish K134/K145 lactylation as indispensable for PRMT1‐mediated cancer cell migration, thereby linking this modification to metastatic progression.

### 2.8 Hypoxia‐Induced HDAC8 Degradation Drives PRMT1 Lactylation and Vimentin Methylation

**Figure 7:**
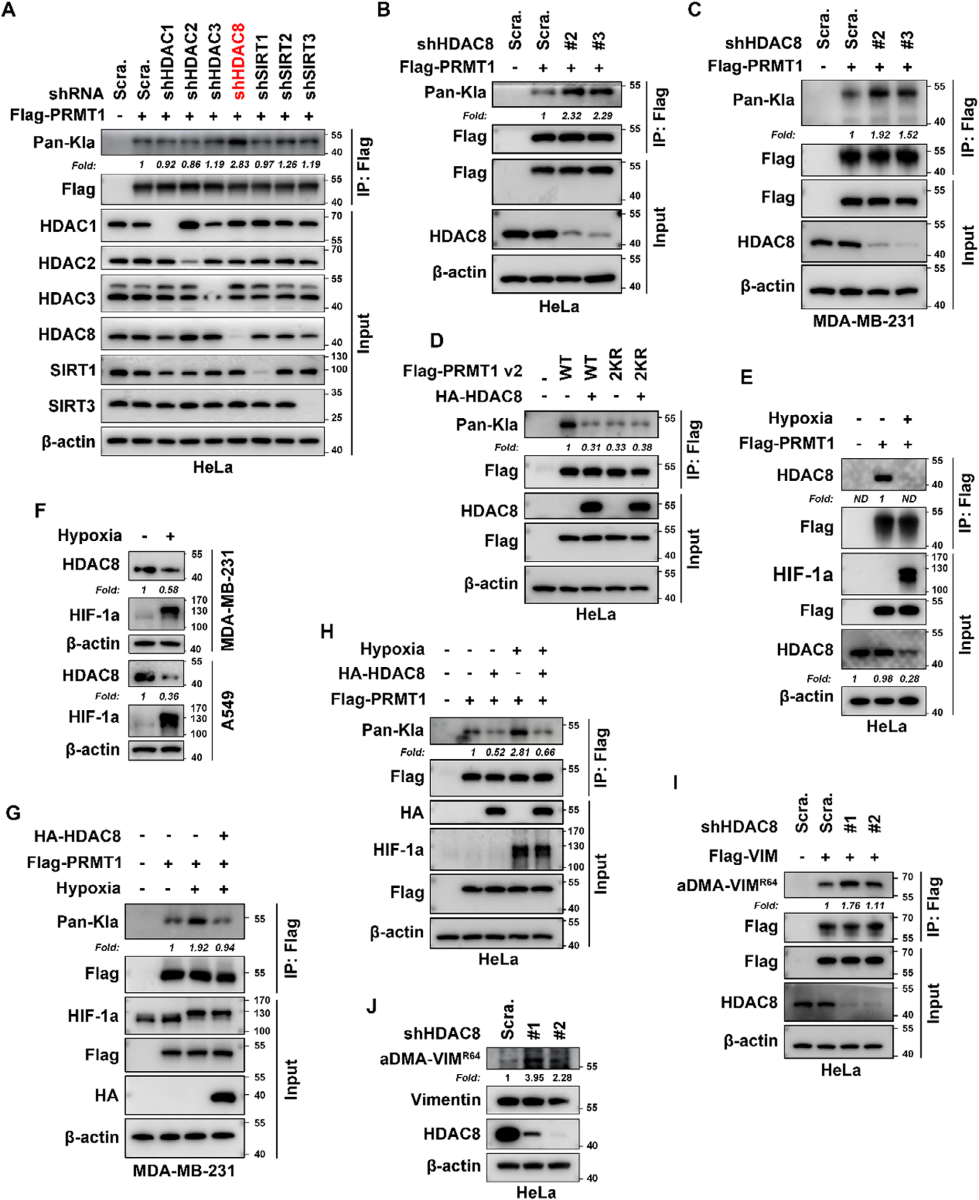
HDAC8 acts as a delactylase for PRMT1 to negatively regulate its lactylation status. A) HDAC8 knockdown selectively increases PRMT1 lactylation in HeLa cells. Cells stably expressing Flag‐PRMT1 were transfected with shRNA‐expressing lentiviruses targeting HDAC1‐3, HDAC8, SIRT1‐3, or non‐targeting shScramble. Seventy‐two hours post‐transfection, lysates were immunoprecipitated with anti‐Flag beads, and PRMT1 lactylation was detected via pan‐lactyllysine (pan‐Kla) antibody, normalized to total Flag‐PRMT1 in IP samples. B,C) Validation of PRMT1 lactylation increase by two additional independent shHDAC8 in HeLa B) and MDA‐MB‐231 C) cells. Cells expressing Flag‐PRMT1 were transduced with two independent shHDAC8 (#2, #3), followed by IP with anti‐Flag beads and pan‐Kla staining, normalized to total Flag‐PRMT1 in IP samples. D) HDAC8 overexpression reduces PRMT1 lactylation. HeLa cells expressing Flag‐PRMT1 WT or 2KR (K134R/K145R) were transduced with HA‐HDAC8 lentivirus or control empty vector. Forty‐eight hours post‐transfection, IP with anti‐Flag beads was performed, and lactylation was detected by pan‐Kla antibody, normalized to total Flag‐PRMT1 in IP samples. E) Hypoxia reduces physical interaction between PRMT1 and HDAC8 in HeLa cells. Cells transduced with Flag‐PRMT1 lentivirus were subjected to IP with anti‐Flag beads, followed by normoxia/hypoxia (1% O2, 12 h) treatment. Bound HDAC8 was probed with anti‐HDAC8 antibody, normalized to immunoprecipitated Flag‐PRMT1 or β‐actin. F) Hypoxia reduces HDAC8 protein levels across cell types. MDA‐MB‐231 and A549 cells treated with normoxia/hypoxia (1% O_2_, 12 h). Whole cell lysates were probed with anti‐HDAC8 antibody, normalized to β‐actin. G,H) HDAC8 overexpression suppresses hypoxia‐induced PRMT1 lactylation. MDA‐MB‐231 G) and HeLa H) cells expressing Flag‐PRMT1 were transduced with HA‐HDAC8 lentivirus, treated with normoxia/hypoxia (1% O_2_, 12 h), and subjected to IP with anti‐Flag beads. Lactylated PRMT1 was detected with pan‐Kla antibody, normalized to total Flag‐PRMT1 in IP samples. HIF‐1α served as a hypoxia marker. I,J) HDAC8 depletion enhances vimentin R64 aDMA. Stable Flag‐vimentin‐expressing HeLa cells I) or HeLa cells J) were transduced with two independent shHDAC8 (#1, #2). Exogenous Flag‐vimentin was immunoprecipitated, and endogenous vimentin was analyzed via Western blot. R64 aDMA levels were detected with aDMA‐VIM^R64^ antibody, and normalized to total vimentin levels in IP or whole cell lysates. All WB experiments were conducted in ≥3 independent biological replicates, and quantified via ImageJ, with fold change relative to control. “*Fold”* = fold change.

To uncover the mechanism underlying hypoxia‐induced PRMT1 lactylation, we built on prior studies showing that histone deacetylases (HDAC1‐3) and sirtuins (SIRT1‐3) mediate protein delactylation.^[^ ^40^ ^]^ We conducted a targeted screen for PRMT1 delactylases by depleting candidate enzymes (HDAC1‐3 and SIRT1‐3) in stable Flag‐PRMT1‐expressing HeLa cells, followed by IP assays to assess lactylation levels (**Figure** **7**A). Notably, depletion of these enzymes did not significantly alter PRMT1 lactylation (Figure 7A). Given a recent report identifying HDAC8 as a potential delactylase – albeit with lower efficiency than HDAC1‐3,^[^ ^41^, ^42^ ^]^ we included HDAC8 in our shRNA screen. Strikingly, HDAC8 knockdown significantly increased PRMT1 lactylation in HeLa cells (Figure 7A). Validation with two additional independent shRNA oligonucleotides confirmed enhanced PRMT1 lactylation in HDAC8‐depleted HeLa cells (Figure 7B), with consistent results in MDA‐MB‐231 cells (Figure 7C), suggesting HDAC8 functions as a bona fide PRMT1 delactylase.

Overexpression of HDAC8 in HeLa cells suppressed lactylation of WT PRMT1, whereas the lactylation‐deficient 2KR (K134R/K145R) mutant remained unaffected (Figure 7D), confirming HDAC8‐mediated regulation of these specific sites. Co‐IP analysis revealed a direct interaction between PRMT1 and HDAC8 under normoxic conditions (Figure 7E), further establishing HDAC8 as the primary delactylase of PRMT1. Notably, hypoxia markedly reduced HDAC8 protein levels, leading to a nearly abolished interaction with PRMT1 (Figure 7E). To determine whether HDAC8 reduction under hypoxia is universal across cell lines, MDA‐MB‐231 and A549 cells were tested, which showed similar decreases in HDAC8 protein levels under hypoxic conditions (Figure 7F). Subsequently, forced HDAC8 expression abrogated hypoxia‐induced PRMT1 lactylation in both MDA‐MB‐231 and HeLa cells (Figure 7G,H), confirming that HDAC8 downregulation is essential for PRMT1 lactylation under hypoxia. In contrast, HDAC8 knockdown increased both exogenous and endogenous vimentin R64 aDMA (Figure 7I,J).

Collectively, these findings establish that hypoxia‐induced HDAC8 downregulation relieves a delactylation‐mediated activation of PRMT1, leading to enhanced K134/K145 lactylation, and subsequent vimentin R64 aDMA.

### 2.9 KAT7 Indirectly Promotes PRMT1 Lactylation and Enhance Vimentin R64 aDMA

To identify the lactyltransferase responsible for PRMT1 lactylation, we conducted a small‐scale shRNA screen in HeLa cells (Figure S9A, Supporting Information). Knockdown of KAT7 significantly reduced hypoxia‐induced PRMT1 lactylation (Figure S9A, Supporting Information), an effect recapitulated by an independent shRNA targeting KAT7 (Figure S9B, Supporting Information). This suppression was selective for wild‐type (WT) PRMT1, as the K134R/K145R (2KR) mutant remained unaffected (Figure S9B, Supporting Information). Conversely, KAT7 overexpression increased lactylation of WT PRMT1 but not its 2KR mutant (Figure S9C, Supporting Information), suggesting that KAT7 may act as a lactyltransferase for PRMT1 at K134/K145. To test this hypothesis, we assessed the physical interactions between KAT7 and PRMT1 via co‐IP assays under normoxia and hypoxia. However, these assays revealed minimal binding between Flag‐PRMT1 and HA‐KAT7 (Figure S9D, Supporting Information), indicating that KAT7 likely regulates PRMT1 lactylation indirectly. Consistent with this model, KAT7 knockdown reduced both exogenous and endogenous vimentin R64 aDMA under both normoxia and hypoxia (Figure S9E,F, Supporting Information). In line with these findings, KAT7 overexpression enhanced endogenous vimentin R64 aDMA under normoxia (Figure S9G, Supporting Information). Collectively, these results demonstrate that KAT7 positively regulates PRMT1 lactylation at K134/K145.

### 2.10 PRMT1 Drives Cancer Cell Migration Through Vimentin R64 Methylation

To determine whether PRMT1‐mediated cancer cell migration requires vimentin, we performed wound healing assays. Vimentin knockdown abrogated the pro‐migratory effect of PRMT1 overexpression in LM2 cells (Figure S10A–C, Supporting Information), indicating that PRMT1 drives cell migration in a vimentin‐dependent manner. To assess whether this effect involves vimentin R64 aDMA, we conducted additional wound healing assays. PRMT1 depletion attenuated the migratory enhancement mediated by wild‐type (WT) vimentin, whereas ectopic expression of the R64F mutant significantly promoted cell migration irrespective of PRMT1 expression levels (Figure S10D–F, Supporting Information). Further analysis showed that PRMT1 overexpression enhanced migration in cells expressing wild‐type (WT) vimentin but had no effect on R64K mutant‐expressing cells. In contrast, cells expressing the R64F mutant exhibited robust motility regardless of PRMT1 expression status (Figure S10G–I, Supporting Information). Collectively, these findings demonstrate that PRMT1 facilitates cancer cell migration through R64 methylation of vimentin.

### 2.11 Genetic Ablation or Pharmacological Inhibition of PRMT1 Disrupts Experimental Metastasis in Xenograft Models

**Figure 8:**
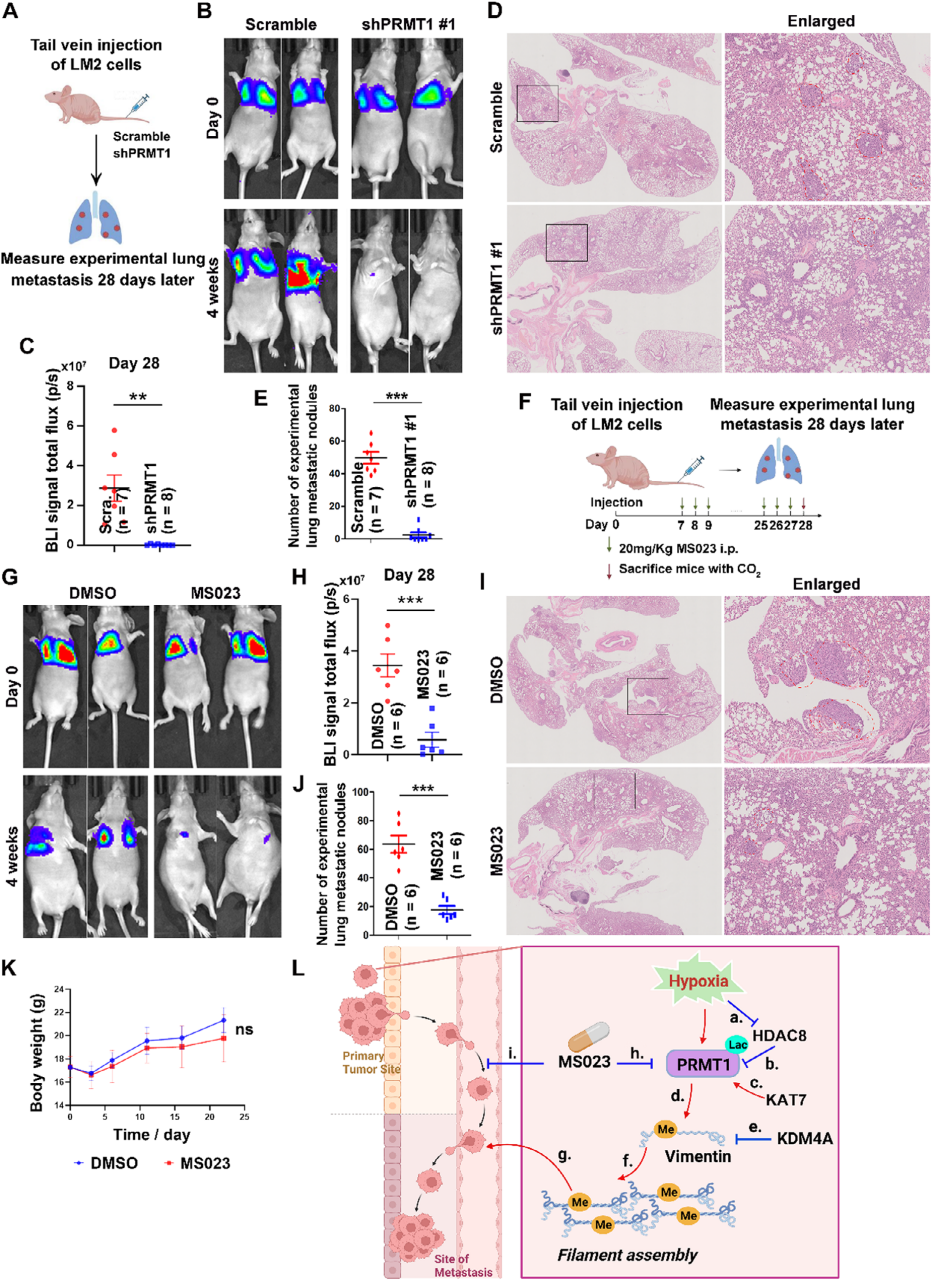
Functional analysis of PRMT1 in experimental metastasis models and evaluation of the therapeutic efficacy of MS023. A–E) In vivo validation of PRMT1‐driven experimental metastasis. A) Stable LM2 cell lines with PRMT1 knockdown (shPRMT1) or control (shScramble) were generated via lentivirus transduction and puromycin selection. B) Female BALB/c nude mice (6‐8 weeks old) were tail‐vein injected with luciferase‐tagged LM2 cells. BLI analysis was performed weekly using an IVIS Spectrum system (PerkinElmer) after intraperitoneal luciferin administration. C) Quantitative analysis of BLI signals at 4 weeks post‐injection (mean ± SEM with n values indicated, two‐tailed unpaired Student's t‐test, **, *p* < 0.01); experiments were terminated at 28 days to allow full phenotypic effects of PRMT1 loss. D) Hematoxylin and eosin (H&E) staining of lung tissues revealed experimental metastatic foci, with magnified regions shown in black boxes. E) Experimental metastatic nodule counts were performed by two independent blinded observers (mean ± SEM with n values indicated, two‐tailed unpaired Student's t‐test, ***, *p* < 0.001). F–K) Therapeutic efficacy of MS023 in experimental lung metastasis. F) Treatment schema: Mice were injected with luciferin‐expressing LM2 cells and treated daily with intraperitoneal MS023 (20 mg kg^−1^ in DMSO) or vehicle control (DMSO) starting 7 days post‐injection for 4 weeks. G) BLI analysis at 4 weeks showed reduced experimental metastatic burden in MS023‐treated mice. H) Quantitative BLI analysis at 4 weeks; experiments terminated at 28 days to allow full manifestation of treatment effects (mean ± SEM with n values indicated, two‐tailed unpaired Student's t‐test, ***, *p* < 0.001). I) H&E staining of lung sections confirmed fewer experimental metastatic foci in MS023‐treated mice, with magnified regions indicated by black boxes. J) Statistical quantification of experimental metastatic nodule number (mean ± SEM with n values indicated, two‐tailed unpaired Student's t‐test, ***, *p* < 0.001). K) Body weight monitoring over 24 days showed no significant difference between groups, indicating lack of severe systemic toxicity (mean ± SEM, two‐tailed unpaired Student's t‐test, ns, *p* > 0.05). L) Proposed mechanistic model of hypoxia‐induced metastatic signaling: Hypoxia triggered HDAC8 degradation a), reducing its delactylation of PRMT1 b). KAT7‐mediated lactylation at PRMT1 K134/K145 c) enhances its methyltransferase activity, promoting vimentin R64 methylation d), which is counteracted by KDM4A e). Methylated vimentin facilitates filament assembly f) and cancer metastatic progression g). MS023 treatment inhibits PRMT1 enzymatic activity h), thereby blocking vimentin methylation and suppressing experimental metastasis i).

To bridge these mechanistic insights into clinical relevance, we generated stable PRMT1‐knockdown LM2 cell lines (shPRMT1) and evaluated their metastatic potential using a mouse xenograft model (**Figure** **8**A). BLI analysis demonstrated that PRMT1 depletion significantly reduced experimental metastatic burden compared to control cells (Figure 8B,C). Histopathological analysis of lung sections corroborated this phenotype, revealing that mice with PRMT1‐depleted tumors harbored markedly fewer and smaller metastatic nodules (Figure 8D,E).

To explore therapeutic implications, we administered the type I PRMT inhibitor MS023 (previously shown to block vimentin R64 aDMA) (Figure 5J; Figure S4L, Supporting Information). Daily intraperitoneal injections of MS023 (20 mg Kg^−1^) potently inhibited experimental metastatic progression, as quantified by BLI analysis (Figure 8F–H) and confirmed by H&E staining of lung tissues (Figure 8I,J). Importantly, MS023 treatment did not induce systemic toxicity, as mice maintained stable body weights throughout the experiment (Figure 8K). Collectively, these data identify the hypoxia‐PRMT1‐vimentin axis as a critical driver of experimental metastatic progression and position MS023 as a promising therapeutic candidate for targeting cancer metastasis (Figure 8L).

## 3 Discussion

This study defines a novel mechanism by which hypoxia enhances PRMT1 activity through lysine lactylation at evolutionarily conserved residues K134/K145 (Figure 7). This post‐translational modification promotes PRMT1‐vimentin interaction and subsequent asymmetric dimethylation (aDMA) of vimentin at arginine (R64), driving cytoskeletal remodeling and metastatic competence in cancer cells (Figure 7). Functional validation using lactylation‐deficient PRMT1 mutants (K134R/K145R) confirmed that this PTM is essential for restoring vimentin filament assembly, and cell migration under normoxia and hypoxia (Figure 7). While histone lactylation has been implicated in hypoxic epigenetic reprogramming,^[^ ^38^ ^]^ our findings extend this concept to protein methyltransferases, establishing PRMT1 lactylation as a redox‐sensitive regulatory switch in metastatic signaling. Notably, PRMT1 lactylation at K145 was independently identified in hepatocellular carcinoma lactylome datasets,^[^ ^28^ ^]^ highlighting evolutionary conservation and pan‐cancer relevance. Although K145 acetylation of PRMT1 has been reported in colorectal cancer (CRC) cells,^[^ ^21^ ^]^ our mass spectrometry analysis detected no acetylation at K134/K145. This mutually exclusive modification pattern points to a critical mechanistic frontier, with implications for uncovering redox‐sensitive nodes that integrate metabolic and epigenetic signaling during tumor progression. We explicitly state that while our data support a role for lactylation at K134/K145, the mutagenesis approach cannot fully exclude contributions from other PTMs. Additional experiments (such as site‐specific lactylation rescue using synthetic peptides or orthogonal editing tools) will be necessary to resolve this ambiguity.

Our shRNA screening identified KAT7 as a candidate lactyltransferase for PRMT1 and HDAC8 as its primary delactylase. While loss‐ and gain‐of‐function experiments demonstrated that KAT7 positively regulates PRMT1 lactylation and downstream vimentin methylation, co‐immunoprecipitation (Co─IP) assays fail to detect a direct physical interaction between KAT7 and PRMT1. This discrepancy raises a key question: does KAT7 mediate PRMT1 lactylation directly (e.g., through substrate scaffolding) or indirectly (e.g., via modulation of metabolic intermediates)? Future studies employing proximity‐based labeling techniques (e.g., proximity‐dependent biotin identification, BioID) or in vitro reconstituted lactylation assays will be essential to resolve this mechanistic ambiguity. In contrast, HDAC8 functions as a non‐canonical delactylase in our model, distinct from the canonical delactylases HDAC1‐3 and SIRT1‐3.^[^ ^22^, ^40^, ^42^ ^]^ Although HDAC8 exhibits substantially weaker delactylase activity compared to HDAC1‐3,^[^ ^41^ ^]^ our data demonstrate that HDAC8 directly interacts with PRMT1 and negatively regulates its lactylation (Figure 7A–G). These findings position HDAC8 as a functional delactylase for PRMT1, yet key questions remain: the scientific community has not reached a consensus on HDAC8's broader role in lactylation regulation, and whether vimentin represents a preferential substrate for HDAC8‐mediated delactylation requires systematic proteomic validation across diverse cellular contexts. Characterizing HDAC8's interactome and substrate landscape under hypoxic versus normoxic conditions will be pivotal to delineating its multifaceted roles in metabolic signaling. Notably, our data reveal that hypoxia induces HDAC8 downregulation across multiple cell lines (Figure 7E,F). The molecular mechanisms governing this downregulation ‐ including transcriptional repression, translational inhibition, or enhanced proteolytic/lysosomal degradation ‐ remain to be clarified. Elucidating how hypoxic stress orchestrates HDAC8 turnover would deepen our understanding of metabolic‐epigenetic crosstalk, as dysregulated delactylase proteostasis may represent a widespread mechanism for amplifying lactylation‐driven oncogenic signaling.

Vimentin's established role in EMT and metastatic dissemination^[^ ^43^ ^]^ contrasts with the incomplete characterization of factors governing its filament plasticity. Here, we identify PRMT1‐mediated asymmetric dimethylation (aDMA) at vimentin R64 as a pivotal regulatory node governing vimentin solubility and filament turnover (Figure 4). The R64K loss‐of‐function mutation abrogates hypoxia‐induced filament assembly, whereas the R64F constitutively methylated mimic drives filament networks formation (Figure 4), establishes vimentin R64 aDMA as a critical activator of its filament assembly. Notably, both hypoxic stress and PRMT1 overexpression robustly enhances vimentin methylation, as evidenced by elevated pan‐aDMA (global asymmetric arginine dimethylation) and R64‐specific methylation signals (Figure 2C; Figure S4I,J, Supporting Information). The disproportionate increase of pan‐aDMA relative to R64‐specific methylation suggests PRMT1 may target additional arginine residues on vimentin for dimethylation (Figure 2C; Figure S4I,J, Supporting Information). While our functional assays establish R64 as a critical determinant of vimentin filament dynamics and cancer cell migration, systematic proteomic mapping of other hypoxia‐PRMT1‐regulated methylation sites on vimentin represents an important future direction. Our findings are supported by orthogonal evidence: BioGRID database analysis confirms PRMT1‐vimentin interactions via affinity purification‐MS,^[^ ^44^, ^45^ ^]^ and prior proteomic studies have implicated vimentin as a PRMT1 substrate.^[^ ^46^ ^]^ In contrast to PRMT5‐mediated symmetric di‐methylation (sDMA) of vimentin at R196/R207/R345/R364 in MTAP‐deficient lung cancer,^[^ ^13^ ^]^ our mass spectrometry analyses detected no dimethylation at these residues under hypoxic conditions, with only mono‐methylation (MMA) observed at R196. These results underscore context‐dependent substrate specificity between PRMT1 and PRMT5, whereby competitive methylation at distinct residues may underlie subtype‐specific metastatic phenotypes. Systematic dissection of methyltransferase crosstalk is therefore indispensable for unraveling the spatiotemporal complexity of cancer progression.

Clinical validation in TNBC specimens reveals that aDMA‐VIM^R64^ (vimentin R64 aDMA) levels correlate with advanced tumor stages and poor patient prognosis, establishing this modification as a potential prognostic biomarker (Figure 3). Pharmacological inhibition of PRMT1 with MS023 (PRMT type I inhibitor) significantly reduces metastatic burden in mouse models (Figure 8), underscoring the therapeutic potential of targeting the hypoxia‐PRMT1‐vimentin axis. However, PRMT1's broad substrate spectrum raises concerns about off‐target effects in vivo. While our data identify vimentin as a critical effector of PRMT1‐mediated metastasis, other known PRMT1 substrates, such as DDX3,^[^ ^47^ ^]^ SRSF1(48), EZH2,^[^ ^49^ ^]^ may also contribute to oncogenic signaling. This warrants further investigation into substrate‐specific roles of these substrates in cancer progression. A notable limitation of this study is the lack of in vivo hypoxia‐mimicking conditions to interrogate PRMT1 function. Although we demonstrate hypoxia‐driven cell migration via the hypoxia‐PRMT1‐vimentin axis in vitro, xenograft mouse models lacking oxygen‐deprived microenvironments may not fully recapitulate the metastatic niche. Future studies could address this by employing combination strategies, such as co‐administration of PRMT1 inhibitors with anti‐angiogenic agents (e.g., bevacizumab^[^ ^50^ ^]^), to induce tumor hypoxia and validate the axis in physiologically relevant contexts. Additionally, while tail‐vein injection models are widely used for assessing post‐intravasation metastasis, they do not capture the full metastatic cascade, including primary tumor EMT and intravasation.^[^ ^51^ ^]^ Incorporating spontaneous metastasis model, in which cancer cells naturally undergo hypoxia‐driven EMT, intravasation, and distant organ seeding,^[^ ^51^ ^]^ would provide more robust mechanistic validation of the hypoxia‐induced PRMT1‐vimentin pathway. Such models would better simulate the clinical metastatic progression, enabling precise dissection of how PRMT1 inhibition affects each stage of the metastatic process under genuine hypoxic stress. Collectively, these insights highlight opportunities to develop substrate‐selective PRMT1 inhibitors and refine preclinical models, bringing us closer to translating these findings into targeted therapies for metastatic cancer.

## 4 Experimental Section

### Cell Cultures and Transfection

The human cell lines A549 (RRID: CVCL_0023), MDA‐MB‐231 (RRID: CVCL_0062), HeLa (RRID: CVCL_0030), HEK293T (RRID: CVCL_0063), and RKO (RRID: CVCL_0504) were originally procured from American Type Culture Collection (ATCC, Manassas, USA). LM2 cells were graciously provided by Dr. Jinquan Liu (Feng Xinhua Laboratory, Life Sciences Institute, Zhejiang University, China).

*Cultivation conditions varied by cell type*: MDA‐MB‐231, HeLa, HEK293T, and LM2 cells were maintained in Dulbecco's Modified Eagle Medium (DMEM; Invitrogen) supplemented with 10% fetal bovine serum (FBS; Gibco) and 1% penicillin‐streptomycin (Gibco). A549 cells required Ham's F12K medium (Invitrogen) with identical supplementations. All cell lines underwent short tandem repeat (STR) profiling for authentication and were confirmed mycoplasma‐negative via PCR‐based detection (MycoAlert Kit, Lonza).

*Model selection rationale*: A549 and MDA‐MB‐231 were chosen as archetypal lung and breast cancer cell lines, respectively, to enable in vitro phenotypic and mechanistic analyses. HeLa cells served as a tractable model system to interrogate the generality of observed phenotypes and mechanisms across diverse molecular contexts. The vimentin‐deficient RKO cell line was employed for ectopic expression studies of vimentin variants, while LM2 cells were utilized to establish xenograft models for in vivo validation. This experimental framework acknowledges triple‐negative breast cancer (TNBC) as the primary clinical context underpinning our initial patient‐derived data analysis.

Standard culture conditions consisted of humidified incubation at 37 °C with 5% CO2 and 21%O_2_ (normoxia). For hypoxic treatments, cells were transferred to a controlled environment chamber maintained at 37 °C, 5% CO_2_, and 1% O_2_. Alternatively, chemical hypoxia was induced by adding 200 µM cobalt chloride (CoCl2; Sigma) to complete growth medium.

Transfection protocols: A549, MDA‐MB‐231, HeLa, RKO, and LM2 cells were transduced using lentiviral vectors (prepared via HEK293T packaging, third‐generation system), whereas HEK293T cells themselves were transfected with polyethyleneimine (PEI; Polyscience) at a 3:1 weight ratio of PET to plasmid DNA.

### DNA Constructions and Mutagenesis

The mammalian expression vector for Flag‐PRMT1‐8, HA‐PRMT1 v5, HA‐PRMT1 v2, Flag‐KDM4A, Flag‐KDM4A^H188A^, GFP‐VIM, HA‐HDAC8, HA‐KAT7 was constructed by inserting the full‐length cDNA into lentiviral expression vector pCDH‐CMV‐EF1‐Puro, with a 3xFlag, 3xHA or GFP tag at the N‐terminus. The mammalian expression vector for Flag‐VIM, HA‐VIM, Flag‐PRMT1 v2 was constructed by inserting the full‐length cDNA into lentiviral expression vector pLVX‐IRES‐Blasticidin, with a 3xFlag or 3xHA tag at the N‐terminus. All the point mutants of PRMT1 v5 (E133Q, E142Q, 2EQ), PRMT1 v2 (K134R, K145R, 2KR), VIM (R64K, R64F), and KDM4A (H188A) were generated using a site‐directed mutagenesis kit (Agilent Technologies). The bacterial expression vector for GST‐PRMT1 v5 was constructed by inserting the full‐length cDNA into pGEX‐6P‐1 (GE Healthcare Life Sciences). For PRMT1 and VIM truncations used in the co‐IP assay, polymerase chain reaction (PCR)‐amplified human PRMT1 and VIM truncations, including PRMT1‐WT, PRMT1‐(△1‐24), PRMT1‐(△25‐343), PRMT1‐(△1‐165), PRMT1‐(△166‐343) subcloned into the lentiviral vector pCDH‐CMV‐EF1‐Puro, fused with a GFP tag in N terminal, and VIM‐WT, VIM‐(△96‐131), VIM‐(△154‐245), VIM‐(△264‐283), and VIM‐(△294‐405) subcloned into the lentiviral vector pCDH‐CMV‐EF1‐Puro, fusing with a 3 x Flag tag in N terminal.

The shRNA vectors were constructed into the pLKO.1‐TRC cloning vector (Addgene, #10 878). The shRNA sequences were as follows:

shPRMT1 #1 (targeting 3′‐UTR): 5′‐TGAGCGTTCCTAGGCGGTTTC‐3′; shPRMT1 #2 (targeting CDS): 5′‐GCAAGTGAAGCGGAATGACTA‐3′; shVIM #1 (targeting 3′‐UTR): 5′‐GCGCAAGATAGATTTGGAATA‐3′; shVIM #1 (targeting CDS): 5′‐GCTAACTACCAAGACACTATT‐3′; shKDM3A #1 (targeting CDS): 5′‐CCCAAGATGTATAATGCTTAT‐3′; shKDM3A #2 (targeting CDS): 5′‐ATCGTGGCACAGTTGCCTAAA‐3′; shKDM4A #1 (targeting CDS): 5′‐CCCGCTTCAAACTGAAATGTA‐3′; shKDM4A #2 (targeting CDS): 5′‐CCGAAACTTCAGTAGATACAT‐3′; shKDM4E #1 (targeting CDS): 5′‐CCCGGTAATCCACCAATTTAT‐3′; shKDM4E #2 (targeting 3′‐UTR): 5′‐CCTCAACAGCACTACTAGTAA‐3′; shKDM5C #1 (targeting CDS): 5′‐GCCACACTTGAGGCCATAATC‐3′; shKDM5C #2 (targeting CDS): 5′‐CCACTACGAACGCATTGTTTA‐3′; shKDM6B #1 (targeting CDS): 5′‐TCTGTACAGACCCTCGAAATC‐3′; shKDM6B #2 (targeting 3′‐UTR): 5′‐AGTCCCACTCACCTCTATTTA‐3′; shJMJD6 #1 (targeting 3′‐UTR): 5′‐CACGGGAACCCATTCACTTAG‐3′; shJMJD6 #2 (targeting CDS): 5′‐GCGGTATGAAAGACCTTACAA‐3′; shHDAC1 #1 (targeting CDS): 5′‐GCCGGTCATGTCCAAAGTAAT‐3′; shHDAC2 #1 (targeting CDS): 5′‐GACGGTATCATTCCATAAATA‐3′; shHDAC3 #1 (targeting CDS): 5′‐CTTTGAGTTCTGCTCGCGTTA‐3′; shHDAC8 #1 (targeting CDS): 5′‐AGTCGCTGGTCCCGGTTTATA‐3′; shHDAC8 #2 (targeting CDS): 5′‐TTACGATTGCGACGGAAATTT‐3′; shHDAC8 #3 (targeting CDS): 5′‐GCGTATTCTCTACGTGGATTT‐3′; shSIRT1 #1 (targeting CDS): 5′‐CCTCGAACAATTCTTAAAGAT‐3′; shSIRT2 #1 (targeting CDS): 5′‐GCCATCTTTGAGATCAGCTAT‐3′; shSIRT3 #1 (targeting CDS): 5′‐GCGGCTCTACACGCAGAACAT‐3′; shKAT2A #1 (targeting CDS): 5′‐GGCTACCTACAAGGTCAATTA‐3′; shKAT2B #1 (targeting CDS): 5′‐TTAATGGGATGTGAGCTAAAT‐3′; shKAT3A #1 (targeting CDS): 5′‐ATCGCCACGTCCCTTAGTAAC‐3′; shKAT3B #1 (targeting CDS): 5′‐ATACTCAGCCGGAGGATATTT‐3′; shKAT5 #1 (targeting CDS): 5′‐CAAGTGTCTTCAGCGTCATTT‐3′; shKAT7 #1 (targeting CDS): 5′‐CGGGATAAGCAGATAGAAGAA‐3′; shKAT7 #2 (targeting CDS): 5′‐TGGAACCGAAGATTCCGATTT‐3′; shKAT8 #1 (targeting CDS): 5′‐CGAAATTGATGCCTGGTATTT‐3′; shAARS1 #1 (targeting CDS): 5′‐GCAGCGATTTATAGATTTCTT‐3′; shAARS2 #1 (targeting CDS): 5′‐CCATCATACCTTCTTTGAAAT‐3′.

### Generation of Stable Cell Lines

Stable cell lines were generated using lentiviruses, including shRNA vectors (targeting PRMT1, VIM, KDM3A, KDM4A, KDM4E, KDM5C, KDM6B, JMJD6, HDAC1‐3, HDAC8, SIRT1‐3, KAT2A, KAT2B, KAT3A, KAT3B, KAT5, KAT7, KAT8, AARS1, AARS2) and overexpression vectors (Flag‐tagged or HA‐tagged PRMT1 v5, PRMT1 v2, VIM, HDAC8, KAT7, KDM4A, PRMT1‐8, KDM3A, KDM4A, KDM4E, KDM5C, KDM6B, JMJD6). Lentiviruses were produced using a two‐plasmid packaging system and transfected into targeted cells. Cells transfected with shRNA‐expressing lentiviruses or pCDH‐CMV‐EF1‐Puro vectors were selected by puromycin (1–2 µg mL^−1^) for 3–7 days, while those transfected with pLVX‐IRES‐Blasticidin vectors were selected with blasticidin (2–5 µg mL^−1^) for 3–7 days.

### Reagents and Antibodies

The following commercially available antibodies were used for Western blotting: β‐actin (Abclonal, #ac026), Vimentin (Abcam, #ab92547), Histone‐H4 (Abcam, #ab10158), Pan‐aDMA (CST, #13 522), Flag (Sigma, #F1804‐1), HIF‐1α (CST, #36169S), HA (Abclonal, #AE105), PRMT1 (Abclonal, #a4502), KDM4A (Abclonal, #a22060), Pan‐Kla (PDMA, #PTM‐1401), HDAC1 (Abcam, #ab7028), HDAC2 (Abclonal, #A22426), HDAC3 (Epitomics, #1580‐1), HDAC8 (Abclonal, #A8865), SIRT1 (Abcam, #ab32441), SIRT3 (Abclonal, #A20805), KAT7 (Abclonal, #A5823).

The anti‐vimentin R64 aDMA‐specific antibody (aDMA‐vimentin^R64^) was prepared by HUABIO (Hangzhou, China). The vimentin (R64, aDMA) peptide (CSPGGVYATR(me, aDMA)SSAVR) was first coupled to keyhole limpet hemocyanin and bovine serum albumin (BSA), followed by multiple subcutaneous injections to immunize New Zealand white rabbits. Subsequently, blood was collected from the rabbits, and the presence of specific antibodies in the serum was evaluated using enzyme‐linked immunosorbent assay (ELISA). For final antibody purification, the serum was passed through the Vimentin (R64, aDMA) peptide column and then passed through a control peptide column to isolate the specific antibody.

The chemical reagents used in this study included 0.1 M CoCl_2_ (Merck, #15 862), D‐Luciferin Potassium Salt (Yeasen Biotechnology, #40902ES03), MS023 (Selleck, #S8112), Puromycin (VWR Life Science, #J593), Blasticidin (Sigma, #SBR00022).

The beads used for IP and pull‐down assays were anti‐FLAG magnetic agarose beads (Thermo Fisher, #A36798), anti‐GFP agarose beads (HUABIO, #NBS01A) and anti‐GST magnetic beads (Beyotime, #P2138).

### Immunoprecipitation (IP) and co‐IP

For immunoprecipitation, cells were lysed using NP‐40 lysis buffer (50 mM Tris‐HCl, pH 7.5; 150 mM NaCl; 1% NP‐40) supplemented with a protease inhibitor cocktail and phosphatase inhibitors (Bimake). The protein lysates were incubated with FLAG beads (Thermo Scientific, #A36798) or GFP beads (HuaBio, #NBS01A) at 4 °C for 2 h. The beads were washed with washing buffer (50 mM Tris‐HCl, pH 7.5; 150 mM NaCl; 0.05% NP‐40), boiled in SDS sample buffer, and then analyzed following a Western blot analysis.

### GST Pull‐Down

Recombinant human GST‐PRMT1 or GST protein purified from *E. coli* was incubated with Flag‐vimentin protein, which was expressed in HEK293T lysate, in NP‐40 lysis buffer at 4 °C for 2 h. Subsequently, the protein mixtures were incubated with GST beads at 4 °C for 1 h. After being washed for three times, the beads were boiled in SDS loading buffer and then subjected to immunoblotting analysis. The GST or GST‐PRMT1 protein was detected by Commassie brilliant blue (CBB) staining, followed by SDS‐PAGE separation.

### Fluorescence Recovery after Photobleaching (FRAP) Assays

HeLa cells were plated in µ‐Dish 35 mm dishes (SAINING, #1 051 000) until they reached the desired density, and then transfected with the GFP‐VIM^wt^ or GFP‐VIM^mt^ plasmids for 48 h. The FRAP assays were performed on a LSM 880 confocal microscope with a thermostatized chamber at 37 °C. Briefly, a pre‐bleach image was captured, after which an area of 5 × 2 µm was bleached using three pulses of 488 nm laser at full power. Post‐bleach single‐section images were acquired every 3 s for 100 s. For fluorescence recovery analysis, the intensity in the bleached region was measured at various time points using the “Frap profiler” plugin in ImageJ. The bleach data were normalized to unbleached regions for all the time points and expressed in arbitrary units in the recovery graphs. A minimum of ten FRAP assays were carried out per experimental condition.

### Vimentin Solubility Fractionation Assay

Vimentin solubility was determined using a previously reported method(30). Briefly, cells were lysed in 5 pellet volumes of ice‐cold RIPA buffer and centrifuged at 12 000 g for 10 min at 4 °C to separate the extract into RIPA‐soluble and RIPA‐insoluble fractions. Prior to centrifugation, 30 µL of RIPA lysates were harvested as whole cell lysates (WCL). Equal volumes of the pellet (30 µL, insoluble vimentin), supernatant (15 µL, soluble vimentin), and WCL (10 µL) were subjected to SDS‐PAGE, and vimentin content was assessed by Western blot analysis. Histone H4 serves as the insoluble control, while β‐actin serves as the loading control for soluble fractions and WCL.

### Identification of Arginine Methylation Sites and Lactylation Sites by Mass Spectrometry Analysis

The arginine methylation sites of vimentin were identified by mass spectrometry (n = 1). Briefly, HEK293T cells stably expressing Flag‐vimentin were lysed and subjected to immunoprecipitation using anti‐FLAG magnetic agarose beads. After being washed with NP‐40 buffer, the Flag‐vimentin protein was collected using SDS buffer, separated by SDS‐PAGE, and stained with Coomassie brilliant blue (CBB).

The lactylation sites of PRMT1 were identified by mass spectrometry analysis under normoxia (n = 1) and hypoxia (n = 1). Briefly, HEK293T cells stably expressing Flag‐PRMT1 (isoform v2) were treated for 12 h under normoxic and hypoxic conditions, respectively. The cells were lysed and subjected to immunoprecipitation using anti‐FLAG magnetic agarose beads. After being washed with NP‐40 buffer, the Flag‐PRMT1 protein was collected by SDS buffer, separated by SDS‐PAGE, and stained with CBB.

The Flag‐tagged vimentin and PRMT1 protein stained by CBB in gels were cut and sent for mass spectrometry analysis at the AIMSMASS Co., Ltd. (Shanghai, China). Detailed information regarding the mass spectrometry analysis was described in the Supporting methods.

### Molecular Docking

The crystal structure of the target protein (PDB ID: 6NT2) was retrieved from the Protein Data Bank. Using PyMOL, non‐native ligands, solvent molecules, and irrelevant polypeptide chains were removed to isolate the functional receptor domain.

Site‐specific modifications were introduced via Python scripting, specifically substituting two lysine residues (Lys134 and Lys145) with lactylated lysine analogs to mimic post‐translational modifications. The lactyl group (─CO─CH(OH)─CH_3_) was added to the ε‐amino group of lysine using established geometric parameters.

Following structural adjustment, protonation states were optimized at pH 7.4 using PROPKA 3.1, and the modified structure was energy‐minimized to ensure conformational stability. High‐resolution renderings of both wild‐type and modified structures were generated in PyMOL, highlighting the spatial arrangement of the lactylated residues and their impact on local protein architecture.

### Animal Studies

The Animal Research Ethics Committee of the Zhejiang University School of Medicine, Sir Run Run Shaw Hospital reviewed and approved the animal study. Experimental lung colonization was assessed via tail vein injection of LM2 cells, a model that evaluates the ability of tumor cells to extravasate and form colonies in the lungs (experimental metastasis) rather than spontaneous metastasis from a primary tumor. LM2 cells with different genotypes (scramble/ shPRMT1) were constructed using the abovementioned methods. Female nude mice (6‐8 weeks old) were purchased from the Shanghai Laboratory Animal Center and housed at the Zhejiang University Laboratory Animal Research Center. Cells were re‐suspended in PBS and 1.5 × 10^5 cells were injected via tail vein into each mouse to establish a lung metastasis tumor model, with 6‐8 mice in each group. Tumor cells in the mice were detected weekly, both on the day of injection and subsequently, using the In Vivo Imaging System (IVIS) in vivo imaging system. Mice were euthanized when their body weight decreased by 20%, dropped below 16 g, or if abnormal symptoms were observed. Mouse lung tissues were then collected for fixation, sectioning, and staining analysis.

Mice were randomly assigned to receive either MS023 or vehicle control (DMSO) (6 mice in each group) one week after tail vein injection. Based on the result of the pilot study, MS023 was administered by intraperitoneal injection at a dose of 10 mg kg^−1^ once daily (the control group was injected with an equal volume of DMSO until mice in the control group reached humane endpoints.

Experimental metastasis assays were terminated at 21 days for genotype‐based comparisons (where phenotypes emerged rapidly) or 28 days for drug/inhibitor studies (to ensure complete penetrance of subtle inhibitory effects).

### Statistics and Reproducibility

All experiments were performed at least three times. The results are presented as the mean ± standard deviation (SD). Statistical analysis was carried out by using GraphPad Prism 8.0. A two‐tailed Student's t‐test was employed to compare variables between two groups. Pearson correlation and linear regression were applied to determine correlations in clinical samples. Survival analysis were analyzed using Kaplan‐Meier survival curves and the log‐rank test. Differences with *p* < 0.05 were considered statistically significant. Statistically significant differences are indicated in the figures and/or figure legends.

## Conflict of Interest

The authors declare no conflict of interest.

## Author Contributions

J.Z., S.Q., X.Y., and Y.W. contributed equally to this work. J.Z., S.Q., X.Y., Y.W., X.Y., H.H., J.L., C.W., L.M., X.L., L.X., J.L., C. W., and Y.P. conducted the experiments and analyzed the data. J.Z., X.C., H.J., and X.W. conceived and supervised the study. J.Z., H.J., and X.W. wrote the manuscript with comments from all authors.

## Ethics Approval

The study was conducted in accordance with the Declaration of Helsinki, and was approved by the Ethics Committee of Sir Run Run Shaw Hospital, the School of Medicine, Zhejiang University (approval number 2024‐2695‐01 and approval date December 12th, 2024). Animal experimental protocols were approved by the animal care committee of Sir Run Run Shaw Hospital, Zhejiang University (SRRSH2025‐0002).

## Supporting information



Supporting Information

## Data Availability

The data that support the findings of this study are available on request from the corresponding author. The data are not publicly available due to privacy or ethical restrictions.
