# Supplementary material for: Hypoxia‐Induced PRMT1 Lactylation Drives Vimentin Arginine Asymmetric Dimethylation in Tumor Metastasis
Source: Adv Sci (Weinh). 2025 Aug 30;12(41):e09861. doi: 10.1002/advs.202509861 (PMC12591124; doi:10.1002/advs.202509861)
Supplement: Supplementary file 1 — Supporting Information [file ADVS-12-e09861-s001.docx]

**Supplementary Materials for**

**Hypoxia-induced PRMT1 lactylation drives vimentin arginine asymmetric dimethylation in tumor metastasis**

Jia Zhou^1,#,^*, Shuying Qiu^1,#^, Xia Yang^2,#^, Yan Wu^1,#^, Xinxia Yao^1^, Hangqi Hu^1^, Jingfeng Luo^1^, Chandra Sugiarto Wijaya^1^, Lingfeng Ma^1^, Xiaojun Long^3^, Lingna Xu^3^, Jinquan Liu^4^, Chaoqun Wang^5^, Yibin Pan^6^, Xiaona Chen^1,^*, Hongchuan Jin^1,^*, Xian Wang^1,^*

Correspondence to: Dr. Xian Wang, [wangx118@zju.edu.cn](mailto:wangx118@zju.edu.cn); Dr. Hongchuan Jin, [jinhc@zju.edu.cn](mailto:jinhc@zju.edu.cn); Dr. Jia Zhou, [zhoujia90@zju.edu.cn](mailto:zhoujia90@zju.edu.cn); and Dr. Xiaona Chen, [cxn@zju.edu.cn](mailto:cxn@zju.edu.cn).

**This PDF file includes:**

Materials and Methods;

Figures S1 to S9;

**Materials and Methods**

**Immunohistochemistry (IHC) staining**

Briefly, paraffin-embedded slides (5 μm) were deparaffinized, rehydrated, and treated with 3% H_2_O_2_ for blocking. Antigen retrieval was carried out using citrate buffer. Subsequently, the sections were blocked with 10% goat serum at 37°C for 1 hour, followed by overnight incubation at 4°C with the specified primary antibody. The slides were then processed using the MaxVision HRP-Polymer anti-mouse/rabbit IHC kit (MXB Biotechnology, 5010), stained with diaminobenzidine (MXB Biotechnology, 0031), and counterstained with Mayer's hematoxylin (MXB Biotechnology, CTS1096). Staining intensity was categorized as negative (0), weak (1), moderate (2), or strong (3). The H-score was computed as (1 × % weak staining) + (2 × % moderate staining) + (3 × % strong staining). Staining intensity and H-scores were independently evaluated by two researchers.

The specificity of the aDMA-VIM^R64^ antibody was first validated in cancer cell lines. Subsequently, two tissue arrays with TNBC clinical samples were then used to evaluate the protein levels of aDMA-vimentin^R64^. One tissue array included 64 clinical samples with tumor tissue and adjacent normal tissues, while the other one consisted of 160 clinical samples containing only triple-negative breast cancer (TNBC) tumors.

**Cell migration assay/ Wound healing assay**

The cell migration ability was evaluated using the cell scratch assay. Cells (5 x 10^5^ cells/ well in 6-well plates) were seeded in 6-well plates. Once the cells adhered to the surface, a sterile 10 μL pipette tip was used to create a scratch in the cell monolayer under sterile conditions with the aid of a straight-edged guide. After scratching, the medium was replaced with medium supplemented with 1% FBS, and the cells were cultured in either a normoxic (21% O_2_) or hypoxic (1% O_2_, 12 hours) incubator. Eighteen fields per well were selected. Images were captured at 0 and 12 hours post-scratching using a low-power objective (10×). Migration data were gathered using ImageJ software, and quantitative analysis of relative open wound area was quantified as (final wound area / initial wound area) × 100% using ImageJ (n=3 independent experiments, 5 fields per well). Cell migration rate, defined as the proportion of wound closure, was calculated as 100% - relative open wound area. Data were derived from three independent experiments with five fields per well, and data processing was standardized across all replicates to ensure consistency.

**Immunofluorescence (IF)**

For immunofluorescence, cells were seeded onto glass coverslips in a 12-well plate, fixed with 4% paraformaldehyde for 25 minutes after washing with PBS. The cells were then incubated for 30 minutes in PBST (PBS containing 0.05% Triton X-100) and 5% BSA, followed by overnight incubation at 4°C with primary antibody. After three washes with PBST, the cells were incubated with an appropriate secondary antibody for 1 hour at room temperature. Finally, the cells were washed again and mounted using a DAPI-containing sealing reagent (Vectorlabs, H-1200). Images were acquired by using a LSM 880 confocal microscope.

**Western blotting**

For immunoblotting, cell samples were lysed in Triton buffer (50 mM Tris-HCl pH 7.4, 150 mM NaCl, 0.5% Triton-X-100) containing a protease inhibitor cocktail (Selleck, #B14001) or RIPA buffer (Beyotime, #P0013D) and then quantified using a BCA protein assay kit (Beyotime, #P0010). The samples were separated by SDS-PAGE and transferred onto PVDF membranes. Primary antibodies were incubated overnight at 4°C. The membranes were then washed with TBST (0.1% Tween-20 in TBS) and incubated with appropriate HRP-conjugated secondary antibodies (Jackson ImmunoResearch). Finally, the membranes were developed with enhanced chemiluminescence (Fudebio, #FD8030) and imaged using the Amersham Imager 600 system (GE Healthcare Life Sciences).

**Sample preparation for mass spectrometry analysis**

The cells were washed with pre-cold PBS, collected and lysed in ST buffer (300mM Tris-HCl pH7.6, 2% SDS), incubated at 95°C for 5 min. Cooled down and centrifuged at 14,000 rpm for 10 min. Pipetted the supernatant to a new tube and reduced with DTT. Mixed 200μg protein with 8M UA in the 10K filter unit and centrifuged at 14,000 g for 15 min. Added 200μL UA and centrifuged at 14,000 g for 15 min. Discarded the flow-through from the collection tube. Alkylated the proteins with IAA and incubated 45 min in the dark. Discarded the flow-through. Added 100μl UA and centrifuged at 14,000 g for 15 min and this step repeat once. Added 200μl 50mM ABC and centrifuged at 14,000 g for 15 min and this step repeat once. Changed a new collection tube and added 4μg trypsin and incubated at 37°C for 16 h. Centrifuged at 14,000 g for 10 min and collected the flow-through to a new tube. Added 50mM ABC and centrifuged at 14,000 g for 10 min, collected the flow-through to the above tube and dried the sample with SpeedVac. The sample was dissolved in 0.1% TFA and desalted with C18 ZipTips and dried with SpeedVac, The sample were resuspended with 0.1% formic acid for mass spectrometry analysis.

**Mass spectrometry analysis**

The peptide samples were analyzed on Thermo Fisher LTQ Orbitrap ETD mass spectrometry, Briefly, samples were loaded onto an HPLC chromatography system named Thermo Fisher Easy-nLC 1000 equipped with a C18 column (1.8mm，0.15 x 100mm). Solvent A contained 0.1% formic acid and solvent B contained 100% acetonitrile. The elution gradient was from 4% to 18% in 182 min, 18% to 90% in 13 min solvent B at a flow rate of 300nL/min. Mass spectrometry analysis was carried out at the AIMSMASS Co.,Ltd.(Shanghai, China) in the positive-ion mode with an automated data-dependent MS/MS analysis with full scans (350-1600 m/z) acquired using FTMS at a mass resolution of 30,000 and the ten most intense precursor ions were selected for MS/MS. The MS/MS was acquired using higher-energy collision dissociation at 35% collision energy at a mass resolution of 15,000.

**Database searching**

Raw MS files were analyzed by MaxQuant (version 1.5.2.8), the parameter used for data analysis included trypsin as the protease with a maximum of two missed cleavages allowed. The mass tolerance for precursor ions and fragment ions was set to 20 ppm and 4.5ppm, respectively. The search included variable modifications of methionine oxidation and deamidation, and fixed modification of carbamidomethylation on cysteine residues. The minimum peptide length was set to six amino acids and a maximum of two miscleavages was allowed. The false discovery rate (FDR) was set to 0.01 for peptide and protein identifications.


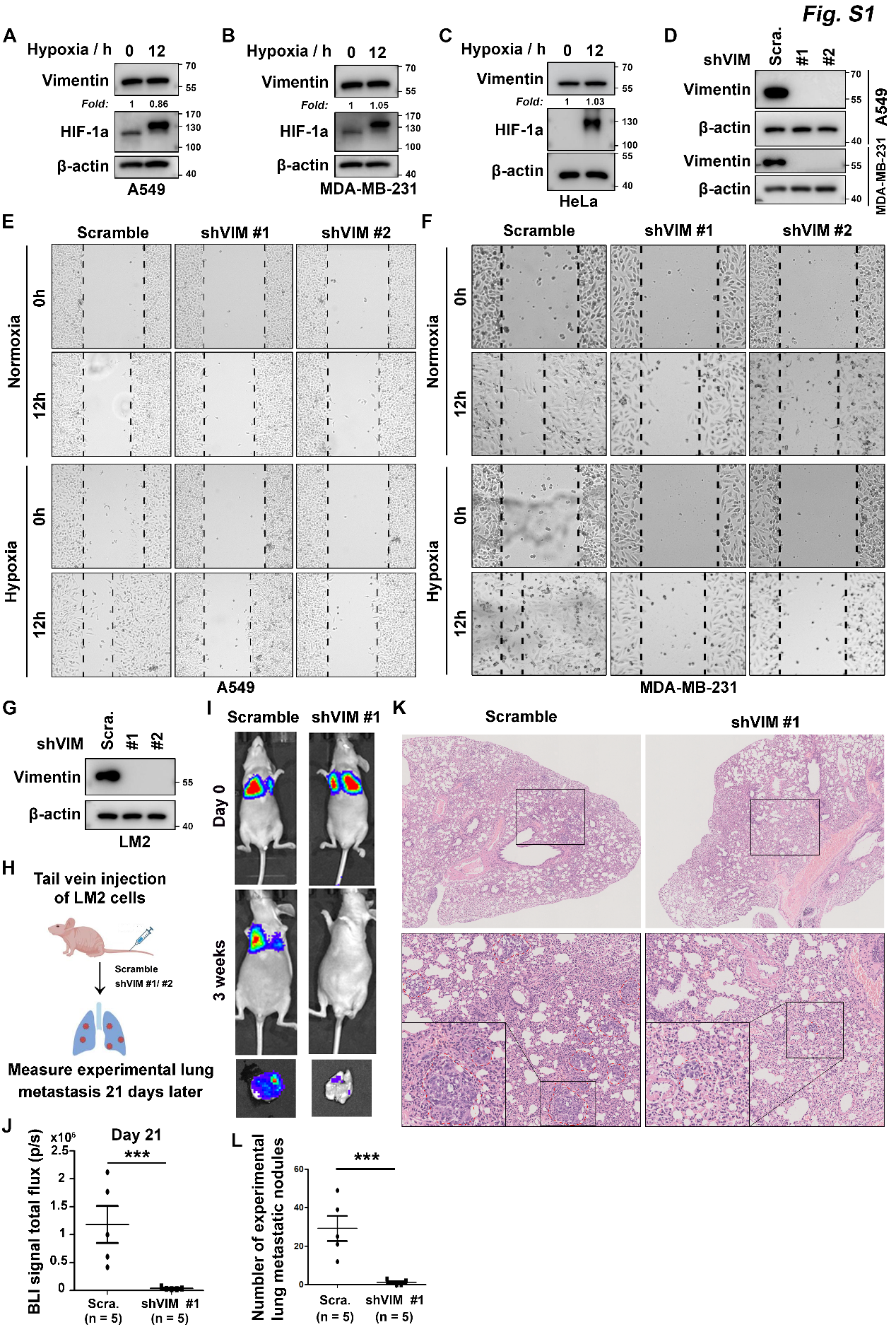


**Figure S1. Loss of VIM inhibits hypoxia-induced cancer cell migration and experimental metastasis.**

**(A-C)** Hypoxic stress does not alter vimentin protein levels. Western blot analysis of vimentin in A549 cells (A), MDA-MB-231 cells (B), and HeLa cells (C) under normoxia (21% O_2_) or hypoxia (1% O_2_, 12 hours). HIF-1α served as a hypoxia marker, and vimentin levels were normalized to β-actin (n=3 independent biological replicates).

**(D)** Validation of vimentin knockdown efficiency. A549 and MDA-MB-231 cells were transduced with shVIM or shScramble lentivirus and selected with puromycin for 72 hours to generate stable knockdown lines. **(E and F)** Wound healing assays in vimentin-knockdown cells. A549 cells (E) and MDA-MB-231 (F) cells with vimentin depletion were subjected to normoxia/hypoxia (12 hours) before scratch-wound analysis. Representative images at 0 and 12 hours post-scratch are shown. **(G)-(L)** In vivo validation of vimentin-mediated experimental lung metastasis. (G) Western blot confirming vimentin knockdown in LM2 stable cell lines (n=3 independent biological replicates). (H) Female BALB/c nude mice (6-8 weeks old) were tail-vein injected with 1.5 x10^5^ luciferase-tagged LM2 cells, and BLI was performed at 4 weeks post-injection using an IVIS Spectrum system (PerkinElmer) after intraperitoneal luciferin administration. shRNA #2 yielded identical migration results in vitro (Figure S1E, F) and was not tested in vivo due to resource constraints; off-target effects were ruled out via wild-type vimentin rescue (Figure 2L). (I) Representative BLI images of experimental lung metastasis. (J) Quantitative BLI analysis of metastatic burden. (K) Hematoxylin and eosin (H&E) staining of lung tissues showing metastatic foci. (L) Statistical quantification of metastatic nodules (n = 5 mice per group).

Western blot band intensities were quantified using ImageJ. Fold change relative to control is indicated as “Fold”. All experiments performed in ≥3 biological replicates unless specified. Data are presented as mean ± SD with n values indicated. Statistical significance determined by two-tailed unpaired Student's *t-*test. ***, *p* < .001.


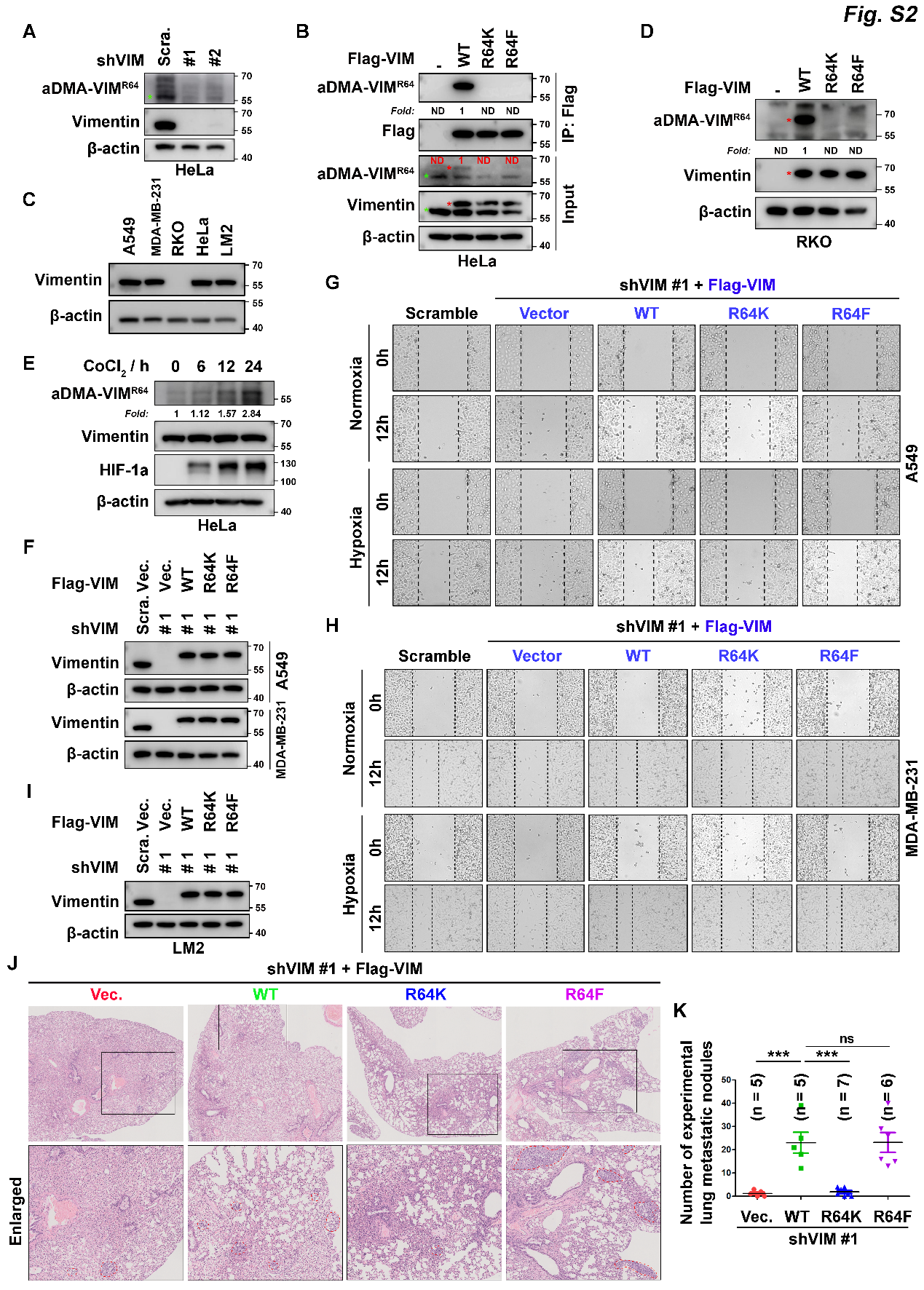


**Figure S2. Validation of aDMA-VIM^R64^ antibody specificity and analysis of vimentin R64 aDMA’s essential role in hypoxia-induced cancer cell migration and experimental metastasis.**

**(A)-(D)** Specificity validation of the aDMA-VIM^R64^ antibody. (A) Western blot analysis of HeLa cells with vimentin knockdown, probed with anti-aDMA-VIM^R64^ and anti-vimentin antibodies. Green star indicates endogenous R64-methylated vimentin signal (abolished in knockdown cells). (B) HeLa cells transduced with Flag-vimentin WT, R64K, or R64F were subjected to immunoprecipitation (IP) with anti-Flag beads. The red star indicated exogenous R64-methylated vimentin detected by aDMA-VIM^R64^ antibody (only in WT), while the red star marks total exogenous vimentin (all Flag-vimentin variants). The green star denotes endogenous R64-methylated/total vimentin (detected in all lanes). (C) Vimentin expression levels in RKO, A549, MDA-MB-231, HeLa, and LM2 cells. Vimentin was undetectable in RKO (vimentin-null) cells. (D) RKO cells reconstituted with Flag-vimentin variants were probed with anti- aDMA-VIM^R64^; anti-vimentin served as a loading control. Methylation levels in (B) and (D) were normalized to total vimentin in IP/whole cell lysates. n=3 independent biological replicates. **(E)** Time-course of hypoxia-mimetic CoCl_2_ treatment on R64 methylation. HeLa cells treated with 200 μM CoCl_2_ for 6-24 hours were analyzed via Western blot. aDMA-VIM^R64^ levels were normalized to vimentin, with HIF-1α as a hypoxia marker (n=3 independent biological replicates). **(F), (I)** Generation of stable vimentin mutant cell lines. A549, MDA-MB-231 (F), and LM2 (I) cells were transduced with shVIM lentivirus, followed by re-expression of Flag-vimentin WT, R64K, or R64F. Stable clones were selected with puromycin/blasticidin and validated via Western blot (anti-vimentin, β-actin loading control, n = 3 independent biological replicates). **(G)-(H)** Wound healing assay with vimentin mutants. A549 (G) and MDA-MB-231 (H) stable lines were subjected to normoxia/hypoxia (1% O_2_, 12 hours) before scratch-wound analysis. Relative open wound closure was quantified as (final wound area / initial wound area) × 100% using ImageJ (n=3 independent experiments, 5 fields per well). **(J)-(K)** Histological analysis of experimental lung metastases. (J) Hematoxylin and eosin (H&E) staining of lung tissues from LM2 vimentin mutant-injected mice, showing representative metastatic foci. (K) Quantitative metastatic nodule counting by two blinded observers (mean ± SD with n values indicated in figure; two-tailed unpaired Student's *t-*test: ns, p > 0.05; ***p < 0.001).​

Western blot band intensities were quantified using ImageJ. Fold change relative to control is indicated as “Fold”; "ND": not detected. All experiments were conducted in ≥3 independent biological replicates unless specified.

**
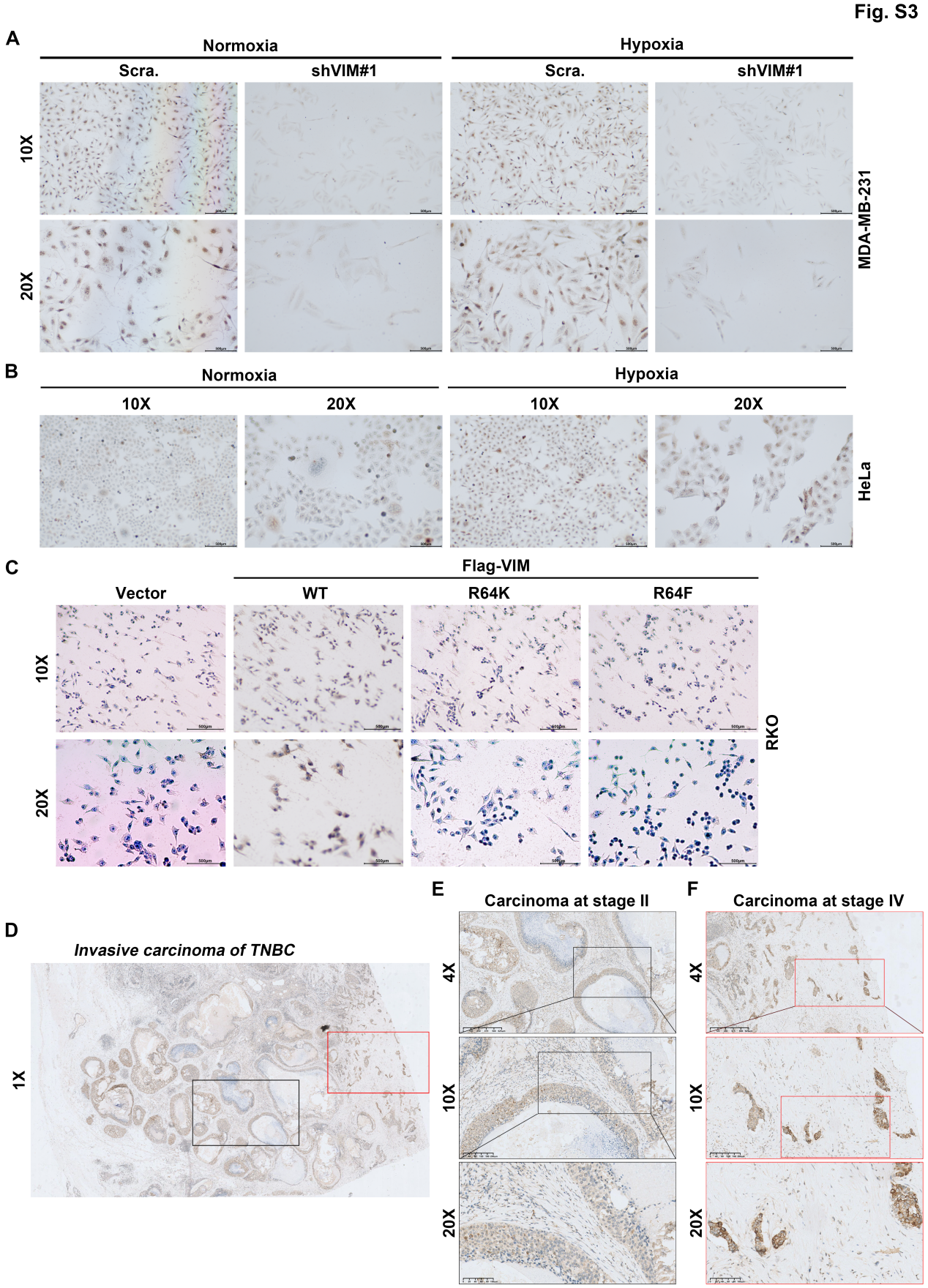
**

**Figure S3. Immunohistochemical (IHC) analysis of aDMA-VIM^R64^ in cancer cell lines and triple-negative breast cancer (TNBC) tissue microarrays.**

**(A)-(C)** Validation of the specificity of the aDMA-VIM^R64^ antibody for IHC applications. (A) IHC staining of MDA-MB-231 cells with vimentin knockdown, cultured under normoxia or hypoxia (1% O_2_, 12 hours), using the anti-aDMA-VIM^R64^ antibody. (B) IHC staining of HeLa cells maintained under normoxia or hypoxia (1% O_2_, 12 hours), probed with anti-aDMA-VIM^R64^ antibody. (C) IHC analysis of RKO cells (a vimentin-deficient cell line) reconstituted with Flag-tagged vimentin variants, using the anti- aDMA-VIM^R64^ antibody. **(D)** Representative IHC staining of aDMA-VIM^R64^ (aDMA-vimentin^R64^) in an invasive TNBC tumor core from a tissue microarray (TMA). Sections were stained with a custom anti-aDMA-VIM^R64^ polyclonal antibody and counterstained with hematoxylin. **(E)-(F)** High-magnification insets (4x, 10x, 20x) of the boxed regions in (D), showing invasive carcinoma areas at different clinical stages: (E) stage II tumor with moderate cytoplasmic aDMA-VIM^R64^ staining (brown signal); (F) stage IV tumor with strong cytoplasmic aDMA-VIM^R64^ expression.


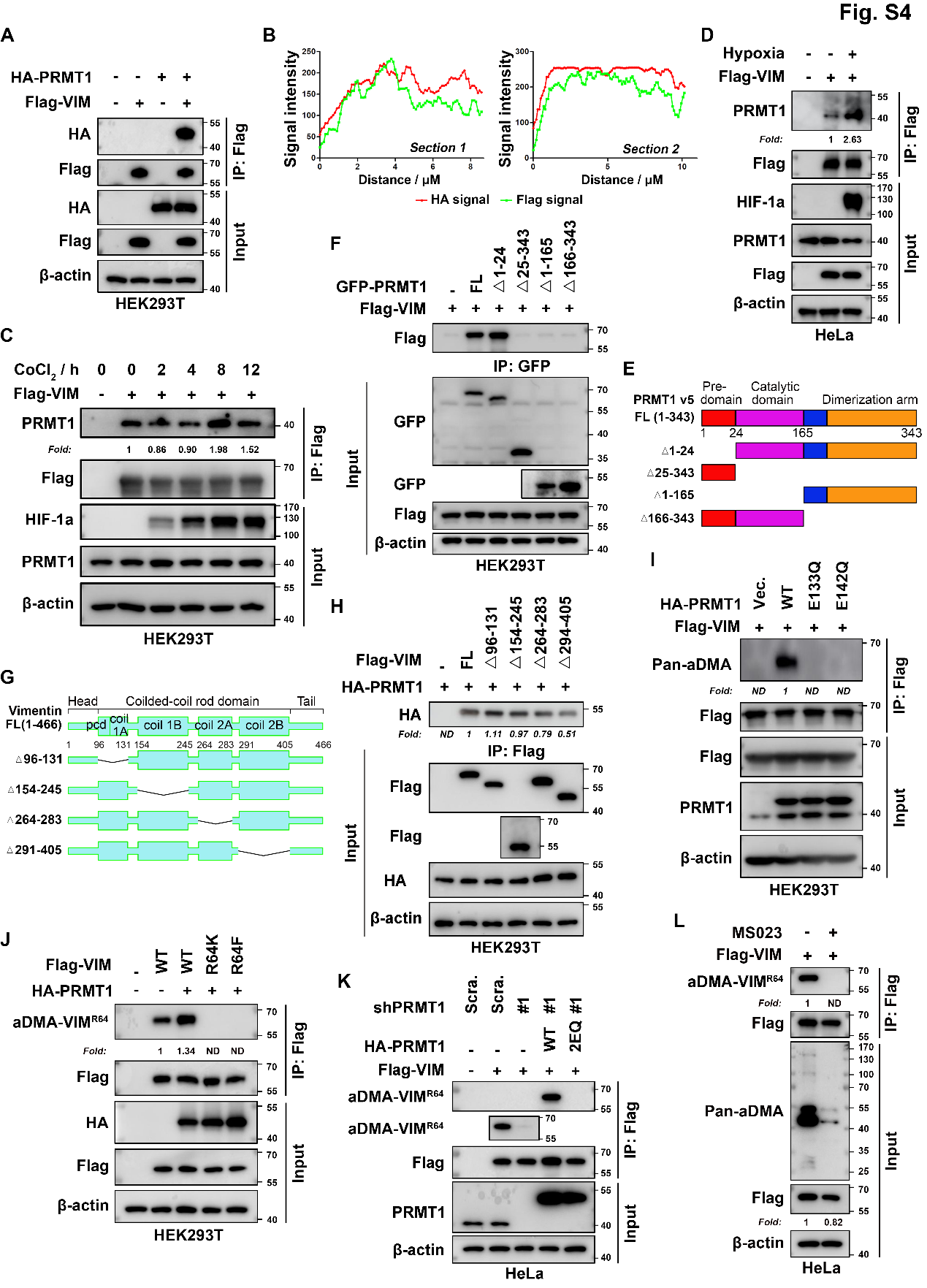


**Figure S4. Characterization of PRMT1-vimentin interaction and regulation of vimentin methylation.**

1. Co-IP validation of PRMT1-vimentin interaction in HEK293T cells. Cells were co-transduced with Flag-vimentin and HA-PRMT1 lentiviruses. At 48 hours post-transfection, lysates were immunoprecipitated with anti-Flag beads and probed with anti-HA and anti-Flag antibodies (n=3 independent biological replicates). **(B)** Quantitative co-localization analysis of Fig.5 C. Pearson’s correlation coefficient was calculated for Flag-vimentin (green) and HA-PRMT1 (red) signals, demonstrating significant cytoplasmic co-localization. **(C)** Time-dependent PRMT1-vimentin interaction under hypoxia-mimetic stress. HEK293T cells expressing Flag-vimentin were treated with 200uM CoCl_2_ for 2-12 hours. Co-IP with anti-Flag beads was performed, and bound endogenous PRMT1 was detected with anti-PRMT1 antibody, normalized to Flag-vimentin in IP samples (n = 3 independent biological replicates). **(D)** Hypoxia-induced PRMT1-vimentin association in HeLa cells. Cells transduced with Flag-vimentin were treated with normoxia/hypoxia (1% O_2_, 12 hours), followed by co-IP and PRMT1 detection as in (C) ( n = 3 independent biological replicates). **(E)-(H)** Domain mapping of PRMT1-vimentin interaction. (E) Schematic of PRMT1 (v5 transcript) truncation mutants. (F) HEK293T cells expressing Flag-vimentin were transfected with GFP-PRMT1 truncation mutants, followed by co-IP with anti-GFP beads and Flag-vimentin detection (n = 3 independent biological replicates). (G) Schematic of vimentin truncation mutants. (H) Cells expressing HA-PRMT1 were transduced with Flag-vimentin truncation mutants, followed by co-IP with anti-Flag beads and HA-PRMT1 detection (n = 3 independent biological replicates). For (F) and (H), exposure times were extended for low-expression mutants (Δ1-165, Δ166-343, Δ154-245) to visualize bands. **(I)** Enzymatically activity-dependent vimentin methylation by PRMT1. HEK293T cells expressing Flag-vimentin were transduced with WT PRMT1, enzymatic inactive mutants (E133Q/E142Q), or control lentivirus. IP with anti-Flag beads was performed, and pan-aDMA was used to detect global aDMA, normalized to Flag-vimentin in IP samples (n=3 independent biological replicates). **(J)** PRMT1 mediates vimentin R64 aDMA inHEK293T cells. Cells expressing Flag-vimentin WT/ R64K/ R64F were transduced with HA-PRMT1 lentivirus. aDMA-VIM^R64^ was detected via IP and custom antibody, normalized to Flag-vimentin in IP samples (n=3 independent biological replicates). **(K)** Rescue of vimentin methylation by PRMT1 catalytic activity. HeLa cells with PRMT1 depletion were reconstituted with WT PRMT1 or 2EQ mutant via retroviral transduction, followed by Flag-vimentin IP (n=3 independent biological replicates). **(L)** Pharmacological inhibition of PRMT1 reduces vimentin R64 aDMA in HeLa cells. Cells expressing Flag-vimentin were treated with 15uM MS023 or DMSO for 24 hours. IP with anti-Flag beads was performed, and aDMA-VIM^R64^ was detected, normalized to Flag-vimentin. Global aDMA levels in input validated drug efficacy (n=3 independent biological replicates).

Western blot band intensities were quantified using ImageJ. Fold change relative to control is indicated as “Fold”; “ND”=not detected. HIF-1α served as a hypoxia marker in (C)-(D). All experiments performed in ≥3 independent biological replicates unless specified.


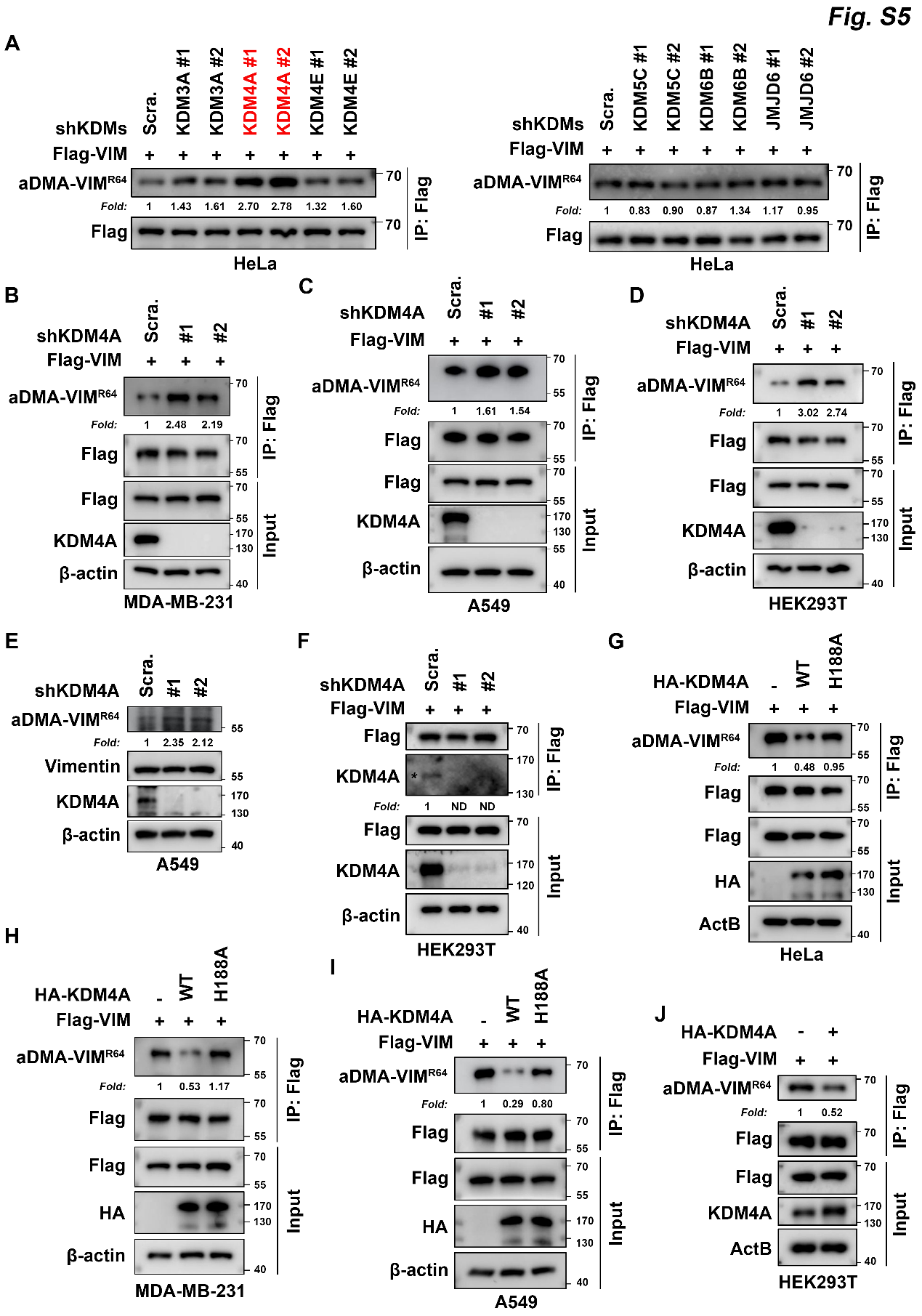


**Figure S5. KDM4A serves as the demethylase for vimentin R64 aDMA.**

**(A)** Screen for vimentin demethylases via a shRNA library. HeLa cells expressing Flag-vimentin were transduced with lentiviruses encoding shRNAs targeting arginine demethylases (KDM3A, KDM4A, KDM4E, KDM5C, KDM6B, JMJD6) or non-targeting shScramble. Seventy-two hours post-transduction, lysates were immunoprecipitated with anti-Flag beads, and R64-methylated vimentin levels were detected using a custom antibody (aDMA-VIM^R64^), normalized to Flag-vimentin (n=3 independent experiments). **(B)-(D)** KDM4A depletion increases vimentin R64 methylation. MDA-MB-231 (B), A549 (C) and HEK293T (D) cells expressing Flag-vimentin were transduced with two independent shKDM4A lentiviruses (#1, #2). At 72 hours post-transduction, IP with anti-Flag beads was performed, and methylated vimentin was detected via aDMA-VIM^R64^ antibody, normalized to Flag-vimentin in IP samples (n=3 independent experiments). **(E)** Endogenous vimentin R64 methylation is enhanced by KDM4A loss in A549 cells. Cells transduced with two independent shKDM4A lentiviruses (#1, #2) were lysed for Western blot analysis, with endogenous R64-methylated vimentin detected using anti-aDMA-VIM^R64^ antibody and normalized to total vimentin (n=3 independent experiments). **(F)** Physical interaction between vimentin and KDM4A in HEK293T cells. Cells expressing Flag-vimentin were transduced with shKDM4A (#1, #2) or shScramble lentiviruses. IP with anti-Flag beads was performed, and bound endogenous KDM4A was detected with anti-KDM4A antibody, relative to control (n=3 independent experiments). **(G-J)** KDM4A overexpression reduces vimentin R64 methylation. HeLa (G), MDA-MB-231 (H), A549 (I), and HEK293T (J) cells expressing Flag-vimentin were transduced with WT KDM4A or enzymatically inactive H188A mutant lentiviruses. At 48 hours post-transduction, IP with anti-Flag beads was performed to detect R64-methylated vimentin with aDMA-VIM^R64^ antibody, normalized to Flag-vimentin in IP samples (n=3 independent experiments).

Western blot band intensities were quantified using ImageJ. Fold change relative to control is indicated as “Fold”. “ND”=not detected.


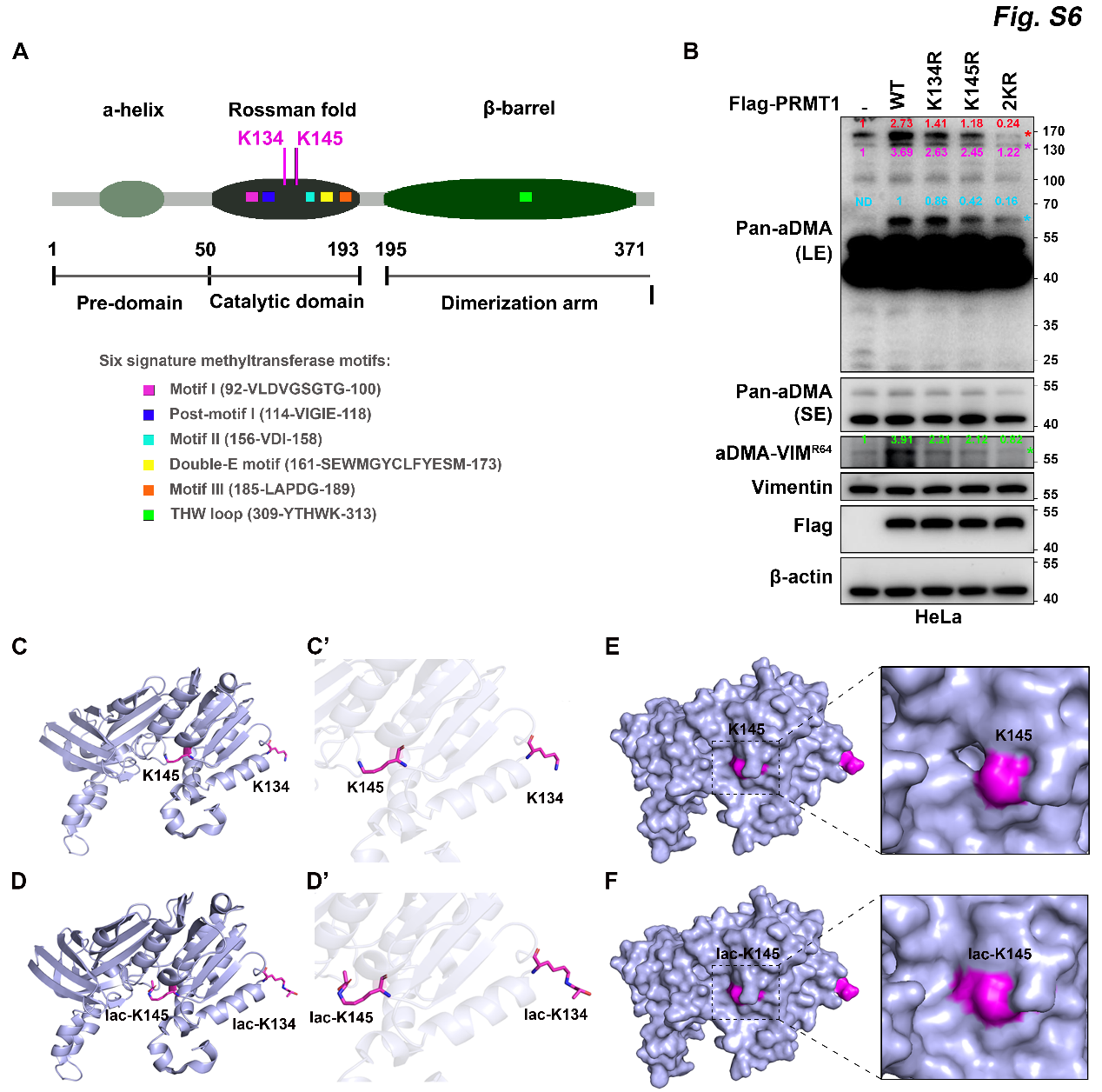


**Figure S6. Domain organization and functional / structural impacts of PRMT1 lactylation at K134/K145 on its enzymatic activity.**

1. Domain architecture of PRMT1 (transcript variant 2). The protein consists of an N-terminal pre-domain (amino acid 1-50), a central catalytic domain (aa 51-193), and a C-terminal dimerization arm domain (aa 195-371). Lysine residues K134 and K145 within the catalytic domain are highlighted in purple. Six signature methyltransferase motifs are denoted by colored boxes. **(B)** Effect of PRMT1 lactylation site mutations on vimentin R64 aDMA and global proteins’ aDMA. HeLa cells transduced with Flag-PRMT1 WT, K134R, K145R, or 2KR (K134R/K145R) lentiviruses were analyzed via Western blot. Whole cell lysates were probed with anti-aDMA-vimentin^R64^ (green star: R64-methylated vimentin, normalized to vimentin) and pan-aDMA antibodies (colored stars: global aDMA, normalized to β-actin). n=3 independent biological replicates. **(C-F)** Structural docking analysis of PRMT1 lactylation at K134/K145. (C-D) Model structures of unmodified PRMT1 (C) and K134/K145-lactylated PRMT1 (D). K134 resides in a flexible loop, while K145 is adjacent to a conserved catalytic domain “channel”. (E) 3D representation of PRMT1 highlighting K134/K145 (pink) and the conserved channel near K145. (F) Lactylated K145 occludes the channel, potentially altering substrate accessibility and catalytic activity.


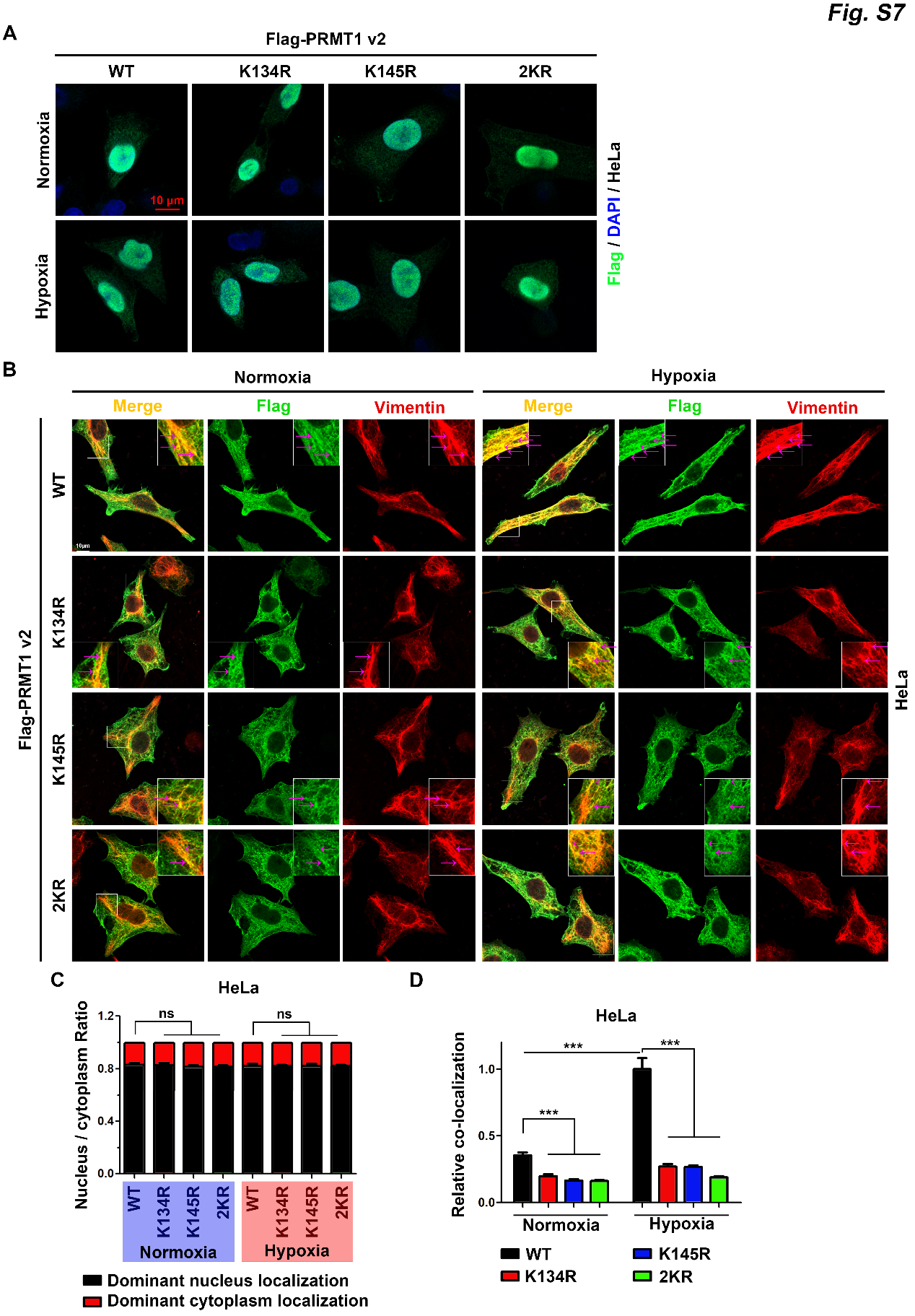


**Figure S7. Immunofluorescence (IF) co-localization analysis of PRMT1 mutants with endogenous vimentin in HeLa cells.**

**(A)-(B)** Representative IF images of Flag-PRMT1 WT, K134R, K145R, or 2KR (K134R/K145R) subcellular localization under normoxia/hypoxia (1% O_2_, 12 hours). Cells were stained with anti-Flag (green) and anti-vimentin (red) antibodies, with DAPI (blue) counterstaining. (A) Nuclear-dominant localization of PRMT1 variants; (B) Cytoplasm-dominant localization of PRMT1 variants. **(C)** Quantitative analysis of PRMT1 subcellular distribution. Percentage of cells with dominant nuclear (A) or cytoplasmic (B) Flag-PRMT1 localization was determined by blind scoring of ≥50 cells per condition across 3 independent experiments. **(D)** Co-localization quantification of Flag-PRMT1 with vimentin. Relative co-localization intensity was calculated as the ratio of co-localized signal area to total vimentin signal area (Relative co-localization = co-localized vimentin area / total vimentin area) using ImageJ (n=5 cells per genotype across 3 independent experiments).

Data are presented as the mean ± SD of ≥3 independent experiments. Statistical significance determined by two-tailed unpaired Student's *t* test. ns, *p* > .05; ***, *p* < .001.


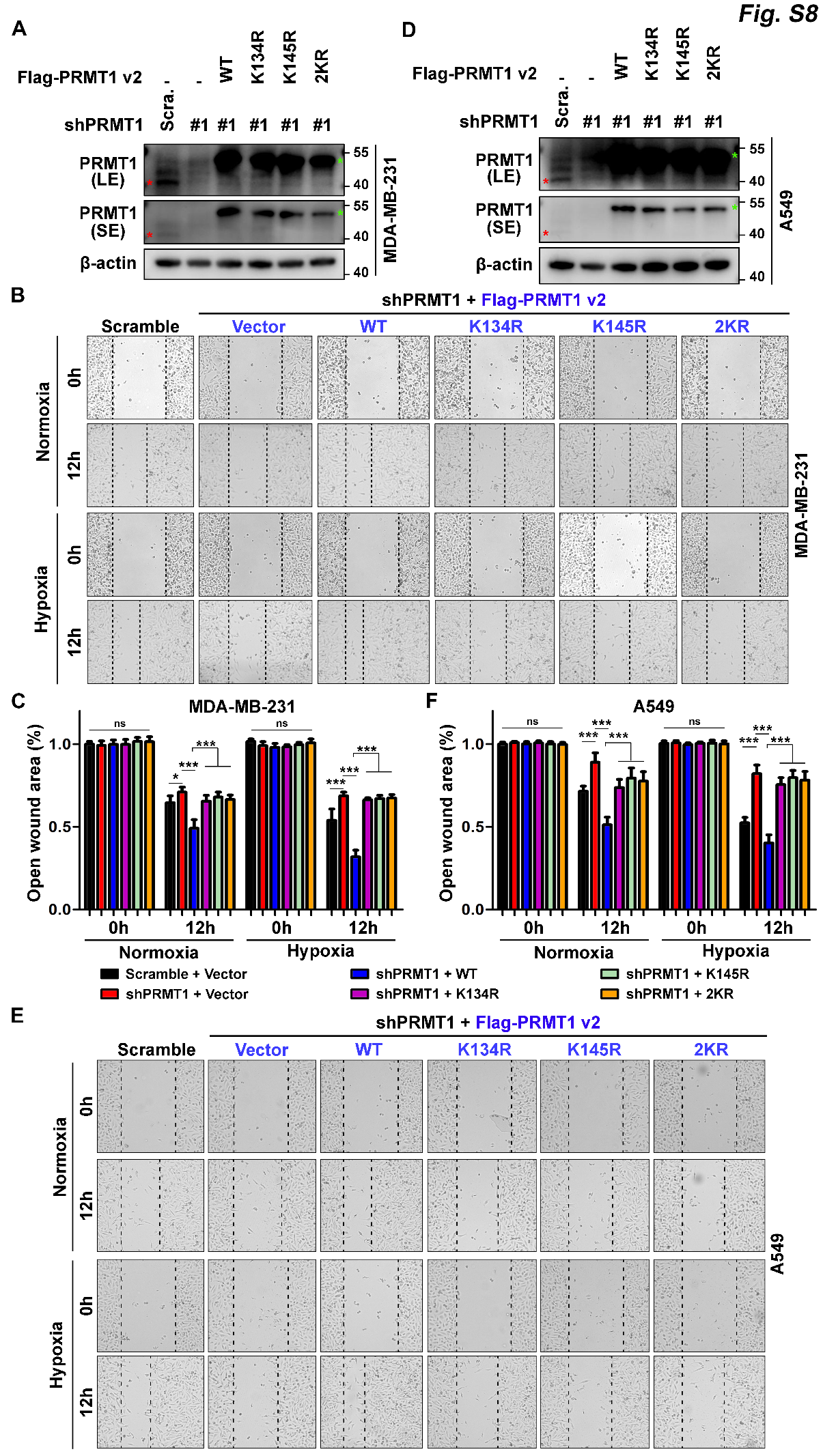


**Figure S8.** **Lactylation of PRMT1 at K134 and K145 contributes to hypoxia-induced cancer cell migration.**

**(A), (D)** Western blot validation of PRMT1 mutant re-expression. MDA-MB-231 (A) and A549 (D) cells were first transduced with shPRMT1 lentivirus, followed by re-expression of Flag-PRMT1 WT, K134R, K145R, or 2KR (K134R/K145R) via retroviral transduction. After puromycin/blasticidin selection, whole cell lysates were probed with anti-PRMT1 antibody, detecting endogenous (red star) and exogenous (green star) PRMT1. “LE” = long exposure; “SE” = short exposure (n=3 independent biological replicates). **(B), (E)** Wound healing assays with PRMT1 mutants. MDA-MB-231 (B) and A549 (E) stable cell lines were subjected to normoxia (21% O_2_) or hypoxia (1% O_2_, 12 hours) before scratch-wound analysis. Representative images at 0 and 12 hours post-scratch are shown. **(C),** **(F)** Quantitative migration analysis. Relative open wound area was quantified as (final wound area / initial wound area) × 100% using ImageJ (n=3 independent experiments, 5 fields per well). Migration rate was calculated as 100% - relative open wound area, with standardized data processing across replicates.

Western blot band intensities were analyzed using ImageJ. Data are presented as mean ± SD of ≥3 independent experiments. Statistical significance determined by two-tailed unpaired Student's *t* test. ns, *p* > .05; *, *p* < .05; ***, *p* < .001.


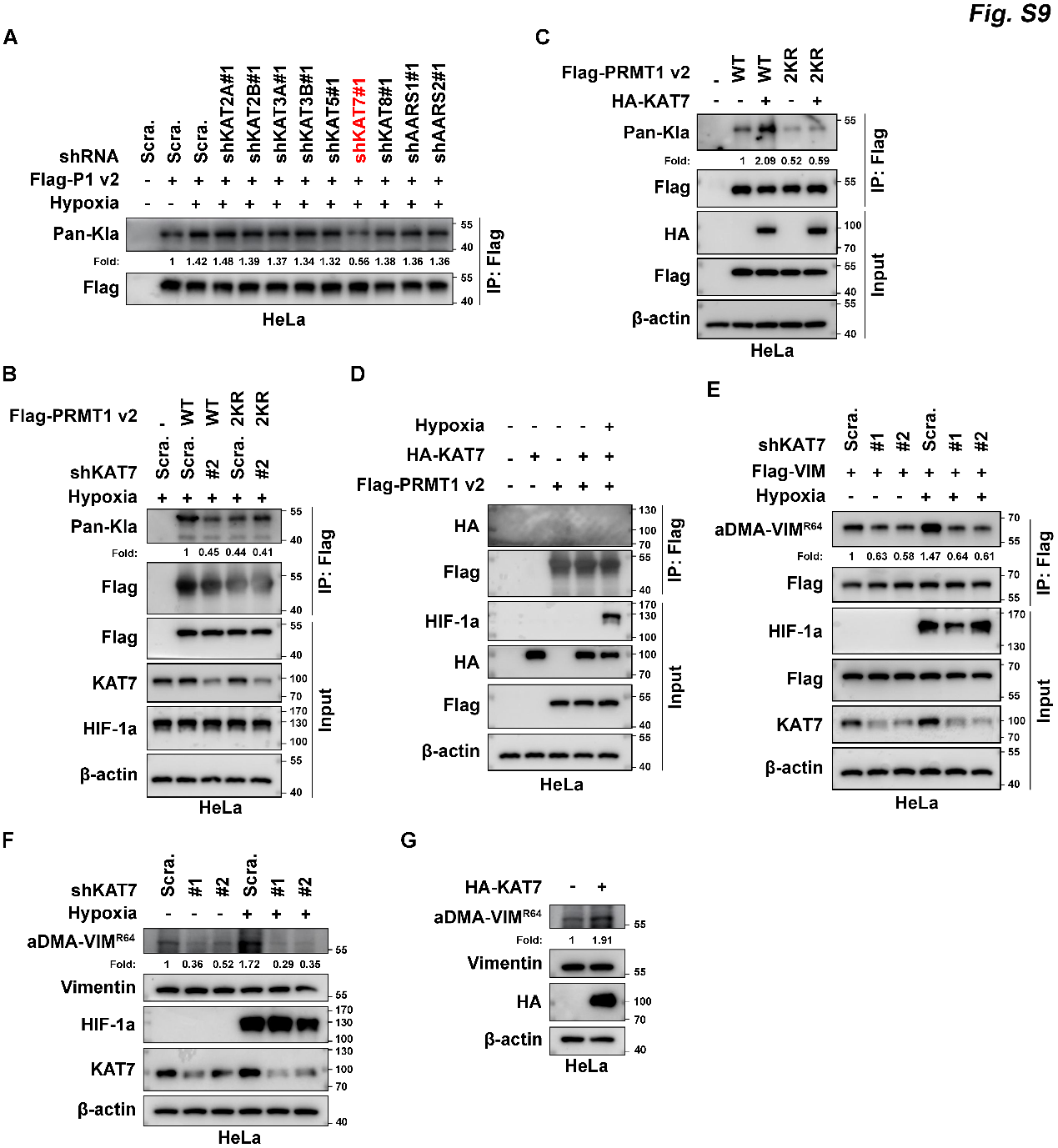


**Fig. S9.** **KAT7 functions as PRMT1 lactyltransferase at K134/K145 and mediates hypoxia-induced vimentin R64 aDMA.**

**(A)** KAT7 depletion selectively reduces hypoxia-induced PRMT1 lactylation. HeLa cells expressing Flag-PRMT1 were transduced with lentiviruses encoding shRNAs targeting candidate lactyltransferases (KAT2A, KAT2B, KAT3A, KAT3B, KAT5, KAT7, KAT8, AARS1, AARS2) or non-targeting shScramble. Seventy-two hours post transduction, cells were subjected to normoxia or hypoxic (1% O_2_, 12 hours), followed by IP with anti-Flag beads. Lactylated PRMT1 was detected using pan-lactyllysine (pan-Kla) antibody, normalized to total Flag-PRMT1 (n=3 independent biological replicates). **(B)** Effect of KAT7 knockdown on PRMT1 lactylation in hypoxic HeLa cells. Cells expressing Flag-PRMT1 WT or 2KR (K134R/ K145R) were transduced with shKAT7 or shScramble, treated with hypoxia (1% O_2_, 12 hours), and subjected to IP. Lactylated PRMT1 was detected using pan-lactyllysine (pan-Kla) antibody, normalized to total Flag-PRMT1 in IP samples (n=3 independent biological replicates). **(C)** KAT7 overexpression enhances PRMT1 lactylation in HeLa cells. Cells expressing Flag-PRMT1 were transduced with HA-KAT7 lentivirus or control vector. After 48 hours post-transduction, IP with anti-Flag beads was performed, and lactylated PRMT1 was detected via pan-lactyllysine (pan-Kla) antibody, normalized to total Flag-PRMT1 in IP samples. Anti-HA staining confirmed KAT7 expression (n=3 independent biological replicates). **(D)** Physical interaction between PRMT1 and KAT7 under normoxia/hypoxia in HeLa cells. Cells co-transduced with HA-KAT7 and Flag-PRMT1 were treated with normoxia (21% O_2_) or hypoxia (1% O_2_, 12 hours), followed by co-IP with anti-Flag beads. Bound HA-KAT7 was detected via anti-HA antibody (n = 3 independent biological replicates). **(E)-(F)** KAT7 depletion reduces vimentin R64 aDMA in HeLa cells. (E) Cells expressing Flag-vimentin were transduced with shKAT7 or shScramble, treated with normoxia (21% O_2_) or hypoxia (1% O_2_, 12 hours), and subjected to IP with anti-Flag magnetic beads. (F) Endogenous vimentin R64 aDMA was analyzed via Western blot. aDMA-VIM^R64^ levels were normalized to total Flag-vimentin (E) in IP samples or total vimentin (F) (n=3 independent biological replicates). **(G)** KAT7 overexpression enhances endogenous vimentin R64 aDMA in HeLa cells. Cells transduced with KAT7 lentivirus or control vector were lysed, and endogenous R64-methylated vimentin was detected via Western blot, normalized to total vimentin (n=3 independent biological replicates).

Western blot band intensities were quantified using ImageJ. Fold change relative to control is indicated as “Fold”.


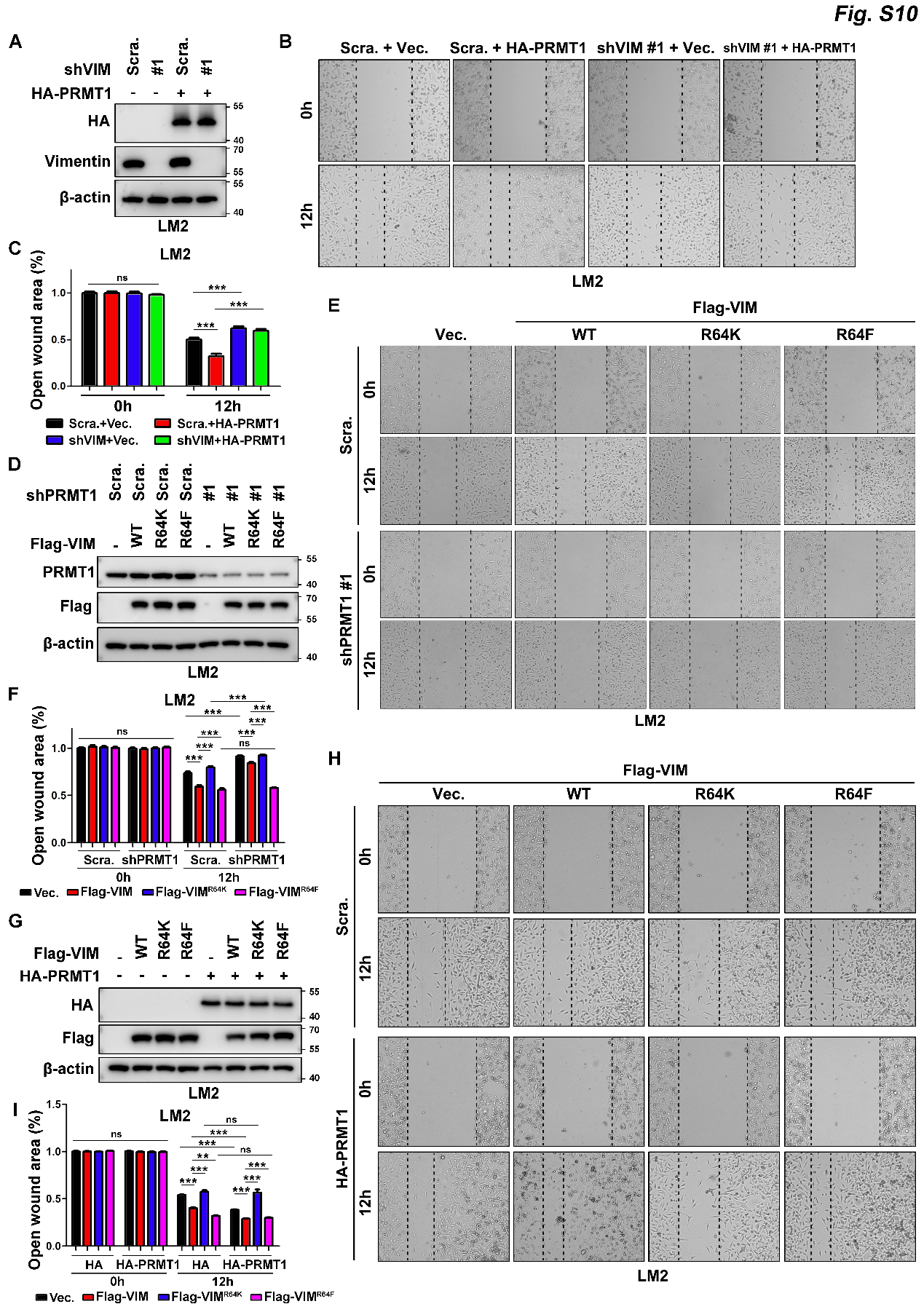


**Fig. S10.** **PRMT1 promotes cancer cell migration through vimentin R64 aDMA.**

**(A)-(C)** Wound healing assay to assess the impact of vimentin loss on PRMT1-promoted cell migration in LM2 cells. (A) Western blot analysis of vimentin knockdown and HA-PRMT1 expression efficiency in LM2 cells. *n = 3 independent biological replicates*.

**(D)-(F)** Wound healing assay to assess the impact of PRMT1 knockdown on the cell migration of LM2 cells expressing Flag-vimentin WT, R64K, or R64F. (D) Western blot analysis of PRMT1 knockdown and Flag-vimentin WT, R64K, R64F expression efficiency in LM2 cells. *n = 3 independent biological replicates*.

**(G)-(I)** Wound healing assay to assess the impact of PRMT1 expression on the cell migration of LM2 cells expressing Flag-vimentin WT, R64K, or R64F. (G) Western blot analysis of HA-PRMT1 and Flag-vimentin WT, R64K, R64F expression efficiency in LM2 cells. *n = 3 independent biological replicates*.

**(B), (E), (H)** Wound healing assays of LM2 cells with indicated genotypes. LM2 cells were cultured under normoxic conditions and assess migratory capacity by scratch-wound analysis. Representative images at o and 12 hours post-scratch are shown.

**(C), (F), (I)** Quantitative migration analysis. Relative open wound area was quantified as (final wound area / initial wound area) × 100% using ImageJ (n=3 independent experiments, 5 fields per well). Cell migration rate, defined as the proportion of wound closure, was calculated as 100% - relative open wound area, with data processing standardized across all experimental replicates to ensure consistency.

All experiments were conducted in at least three independent biological replicates, unless otherwise specified. All data shown in the figure are presented as the mean ± SD of at least three independent experiments. The *p* value was determined by two‐tailed unpaired Student's *t* test. ns, not significant (*p* > .05); ***, *p* < .001.
